# Supplementary material for: Electronic transport in planar atomic-scale structures measured by two-probe scanning tunneling spectroscopy
Source: Nat Commun. 2019 Apr 5;10:1573. doi: 10.1038/s41467-019-09315-6 (PMC6450957; doi:10.1038/s41467-019-09315-6)
Supplement: Supplementary file 1 — Supplementary Information [file 41467_2019_9315_MOESM1_ESM.pdf]

Supplementary Information for:

Kolmer M. et al., **Electronic transport in planar atomic-scale structures measured by two-probe scanning tunneling spectroscopy**

Supplementary Note 1: Two-probe STM/STS experiment methodology

In order to establish two-probe scanning tunneling microscopy (2P-STM) experiments on atomically defined positions we follow previously reported methodology[1], which includes three main steps:

Coarse positioning of STM probes navigated by scanning electron microscope (SEM). We approach both probes with STM feedback on the Ge(001) surface. After approach the lateral distance between probe apexes is about 2 µm. The positioning is performed under SEM in order to control lateral positions of probes in µm scale. Usage of SEM during this inevitable technical step affects the final atomic-scale quality of a surface area investigated in 2P-STM experiments due to presence of SEM-induced defects on germanium surface[1].

Large scan STM imaging. We perform series of STM images until the same surface area is sequentially observed by both STM probes in order to establish their relative positions with respect to the Ge(001) surface.

Atomic scale positioning. Chosen area of the Ge(001) surface is sequentially imaged with atomic resolution by both STM probes in order to establish their positions with respect to the atomic reconstruction of the Ge(001)-c(4×2) surface. Then simultaneous STM imaging performed on both probes gives the exact lateral STM apex-to-apex distance used in two-probe scanning tunneling spectroscopy (2P-STS) experiments. Here we worked on apex-to-apex distances

down to about 30 nm.

To establish 2P-STS experiment we perform the following procedure:

Two lock-in amplifier setup. AC component (∼30 mV peak to peak, 680 Hz) is added to DC bias voltage on the source probe (tip1). Tunneling current signals from source (tip1) and drain (tip2) probe are then demodulated at this given frequency, what results in corresponding dI_1/dV_1 (vertical, differential conductance) and dI_2/dV_1 (planar, differential transconductance) STS signals. Phases of demodulation are chosen with respect to the same AC reference component for both probes.

Drain probe position and contact. For a chosen location of tip2 we follow the previously described procedure of tip-sample contact resistance determination[1]. In this work we typically start from z_0 value determined by (-0.5 V, 20 pA), switch off STM feedback loop, apply virtual ground bias voltage (typically lower than ±1 mV as determined by I=0 nA value on I-V curve) and approach tip2 towards the sample by 0.2-0.5 nm. The resulting tunneling resistance was typically in the range of 10-1000 MΩ (see **Supplementary Figure 1)**. In this work depending on exact tip apex we tried to keep the largest possible resistance that gave us detectable and stable I_2, dI_2/dV_1 signals during 2P-STS operation. During 2P-STS experiment the feedback loop is switched off and the drain probe is maintained at constant height with the predefined tunneling junction resistance.

Source probe positioning and 2P-STS. During 2P-STS experiments source probe (tip1) is maintained in tunneling regime (-0.5 V,

10-100 pA). Positions of tip1 are determined by imaging performed with STM feedback loop. The STS experiment is realized in a standard sequential manner: Tip1 is moved to a chosen position; Feedback loop is switched off; Bias voltage on source probe is ramped (∼100-150 s total acquisition time) and corresponding I_1, I_2, dI_1/dV_1 and dI_2/dV_1 signals are registered; Feedback loop is turned on and the procedure can be repeated. Note that despite the grounded sample, a given DC bias applied on the source probe (also without AC component) results in detection of a current signal on the drain probe pre-amplifier only if both tips are located tens of nanometers apart on the same reconstructed Ge(001) terrace. The same procedure applied on Au(111) surface at similar experimental conditions (∼4.5 K) does not give detectable I_2 and dI_2/dV_1 transconductance signals on distances down to 50 nm.

Supplementary Note 2: Probe to sample contacts

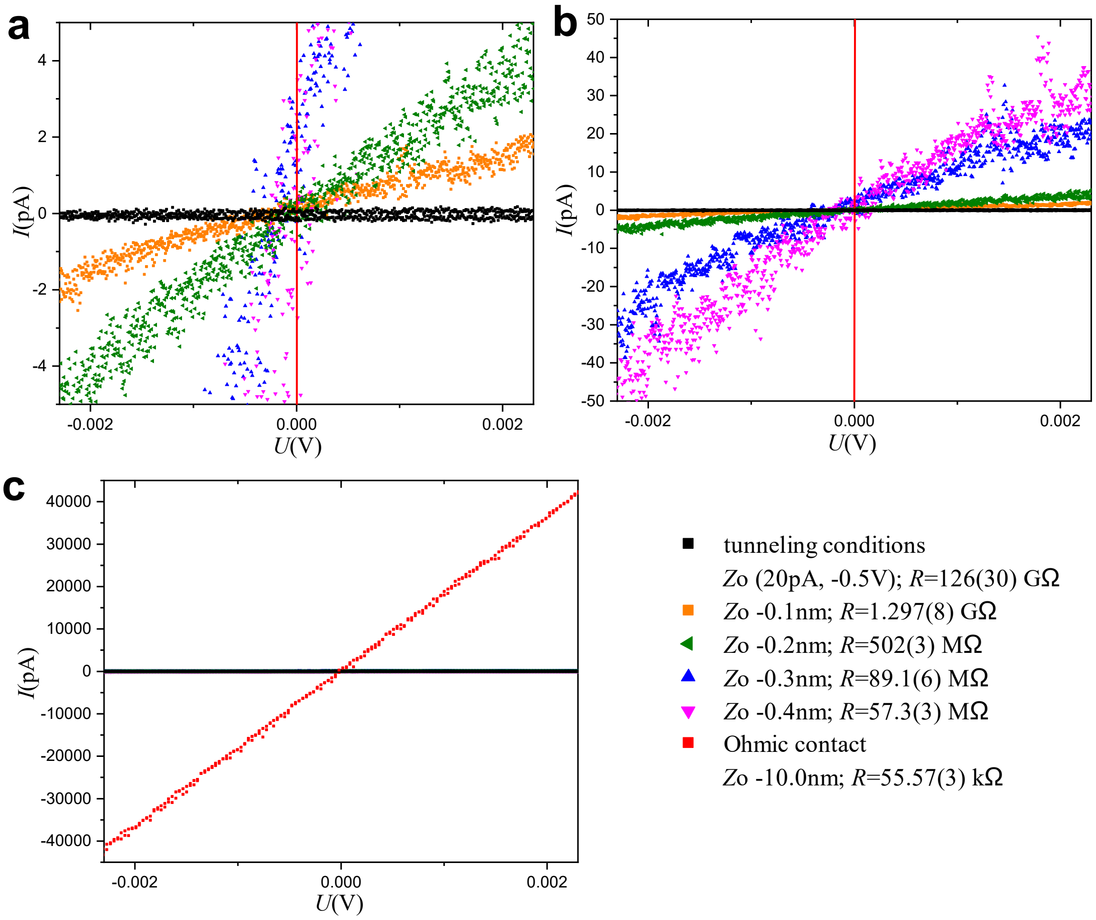

Supplementary Figure 1. **Characterization of the contact.** The contact between the PtIr scanning tunneling microscopy (STM) tip and the Ge(001) surface is presented for different tip-surface distances at 4.5 K. Graphs in **(a-c)** present the same sets of data at three current regimes. For each tip-sample distance we present two sets of raw data. The $I-V$ characteristics around Fermi energy show linear dependence due to the Ge(001) surface Fermi level pinning effect[1–4]. Starting from high resistance tunneling conditions $Z_0(-0.5$ V, 20 pA, black points) the tip is progressively approached towards the surface by 0.1 (orange), 0.2 (green), 0.3 (blue), 0.4 (pink) and 10 nm (red), what changes the corresponding junction resistances from above 100 GΩ (pure tunneling conditions) to about 50 kΩ (saturated value of resistance reflecting multi-channel Ohmic contact). Note that standard two and four probe experiments are performed in the regime of Ohmic contacts, what hinders the understanding of the atomic-scale processes behind the electronic transport at the tip-sample junction. Also note that such a low resistance Ohmic contact requires a large contact area and a strong interaction between tip and surface that is likely to cause strong non-controllable modifications on the surface structure.

Supplementary Note 3: Additional two-probe STS data

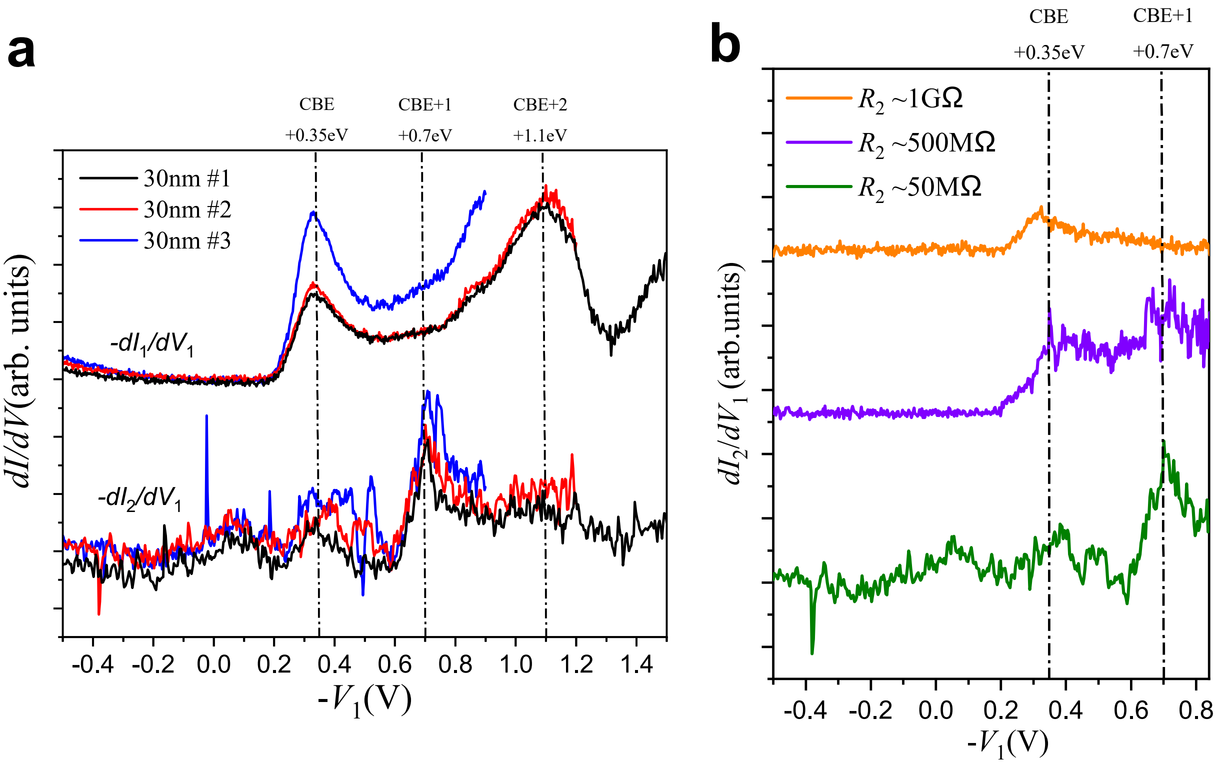

Supplementary Figure 2. **Two-probe scanning tunneling spectroscopy (2P-STS). Contact resistance dependence.** **(a)** Set of three 2P-STS) data obtained by probes positioned on the same Ge dimer row on c(4×2) reconstructed Ge(001) for probe-to-probe distance of 30 nm. Three pairs of simultaneously obtained vertical $-dI_1/dV_1$ and planar transconductance dI_2/dV_1 signals as a function of tip1 voltage are shown by black, red and blue curves respectively. The resonances observed in the dI/dV characteristics at energies 0.35 eV, 0.7 eV and 1.1 eV are ascribed to surface conduction band edge (CBE), CBE+1 and CBE+2, respectively. Data were obtained with the application of the protocol with two lock-in amplifiers at the same experiment conditions as discussed in the main text (**Fig. 2c**), excluding closer tip1-sample Z_0 distance for blue spectra, which was defined by V_{sample}=-0.5 V and I=20 pA (instead of 10 pA for other data). Note that about two times lower tunneling resistance of tip1-sample junction does not affect the general characteristics of transconductance signal. **(b)** Set of three 2P-STS transconductance data as a function of tip1 voltage obtained by probes positioned on the same Ge dimer row on c(4×2) reconstructed Ge(001) for different values of tip2-surface low-bias resistance (see legend). tip1-surface distance was defined by V_{sample}=-0.5 V and I=10 pA. The relative distance between probes was 30 nm for black and 37 nm for red and blue spectra. Note that relative intensities of resonances in transconductance dI_2/dV_1 signals reflect similar trend to calculated spectra presented in **Supplementary Figure 11e,f**.

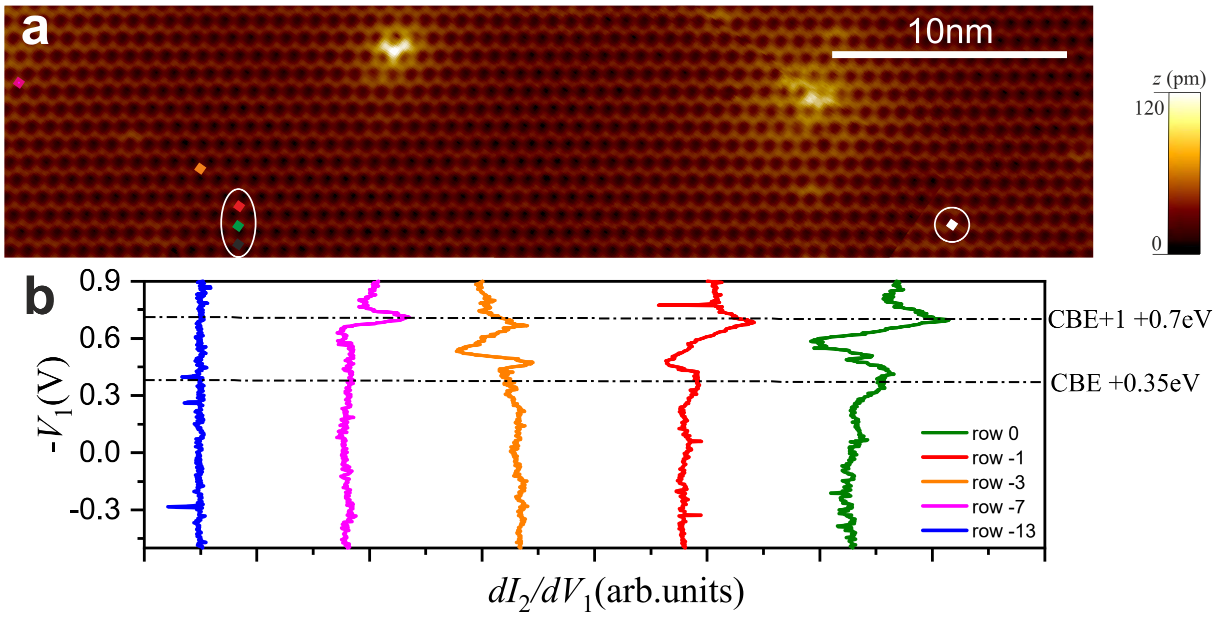

Supplementary Figure 3. Two-probe scanning tunneling spectroscopy (2P-STS). Characterization of c(4×2) reconstructed Ge(001). **(a)** Filled-state scanning tunneling microscopy (STM) image (V_{sample}=-0.5 V, I=20 pA) obtained prior to the 2P-STS experiment. STM tip1 positions during 2P-STS are marked by different color squares (white ellipse marks points from **Fig.5 a,b**). Position of tip2 is marked by white circle. The increase of probe-to-probe distance during characterization of different relative rows is due to probe geometric constrains. **(b)** Planar transconductance dI_2/dV_1 2P-STS signals as a function of tip1 voltage obtained for STM probes located at different reconstruction rows (see **a**, blue reference spectrum was obtained outside the presented image). The corresponding rows of separation are indicated on the label (0 is the same Ge dimer row). During acquisition of data the sample was grounded and the tunneling contact resistance of the tip2-sample junction was established at ∼50 MΩ and kept constant. tip1-sample distance was established in all cases at Z_0 defined by V_{sample}=-0.5 V and I=20 pA. We observe strong suppression of transconductance dI_2/dV_1 resonance at 0.35 eV while increasing separation across reconstruction rows between injection and detection of carriers, what confirms its quasi-1D character. The 0.7 eV resonance was in this case still preserved even at 7 rows of separation.

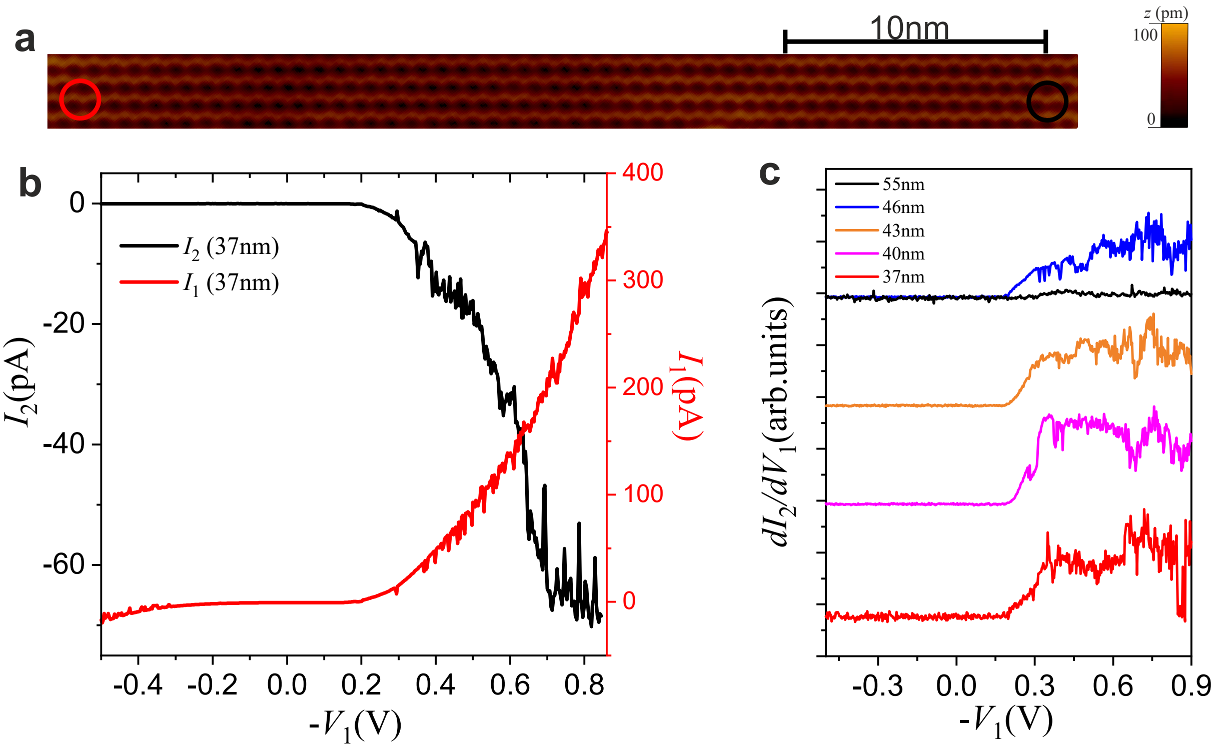

Supplementary Figure 4. Two-probe scanning tunneling spectroscopy (2P-STS). Characterization of a Ge single dimer row on c(4×2) reconstructed Ge(001). (**a**) Filled-state STM image (V_{sample}=-0.5 V, I=50 pA) obtained prior to the 2P-STS experiment. STM probe positions for 2P-STS about 37 nm apart over the very same Ge dimer row are marked by red (tip1) and black (tip2) circles. (**b**) Current versus tip1 voltage curves obtained simultaneously on source (tip1, red) and drain (tip2, black) probes during 2P-STS experiment performed with tips placed in positions marked in **a**. The sample was grounded and the tunneling contact resistance of tip2-sample junction was established to be ∼500 MΩ. (**c**) Transconductance dI_2/dV_1 2P-STS data as a function of tip1 voltage obtained at the same Ge reconstruction row at different tip to tip distances. The red curve was obtained with tips placed in positions marked in **a**. The signature of resonances starts to be detected at probe to probe separation distance about 50 nm. Such value is in perfect agreement with the coherence length of quasi-particles of that energy obtained from the step-edge reflection experiment. Note that these experiments were realized with a different pair of STM probes than the one shown in the main text (**Fig. 2** and **Supplementary Figure 3**) and the resonances observed in the transconductance dI_2/dV_1 signals are found at energies around 0.35 eV (CBE) and 0.7 eV (CBE+1).

Supplementary Note 4: Capacitive coupling between closely spaced metallic probes

For the lock-in detection technique we used additional AC bias applied to tip1. This may induce artificial signal detection on the demodulator of the tip2 current signal due to possible AC coupling between closely spaced STM probes. However, this effect is not affecting general results in the presented experiment, as the direct correspondence of measured dI_2/dV_1 to differential conductance may be proved by reproduction of the signal from DC current by its numerical differentiation (see **Supplementary Figure 5**). Finally, the transconductance dI_2/dV_1 resonances were not registered for both probes kept closely spaced laterally if the drain probe was slightly retracted from the surface.

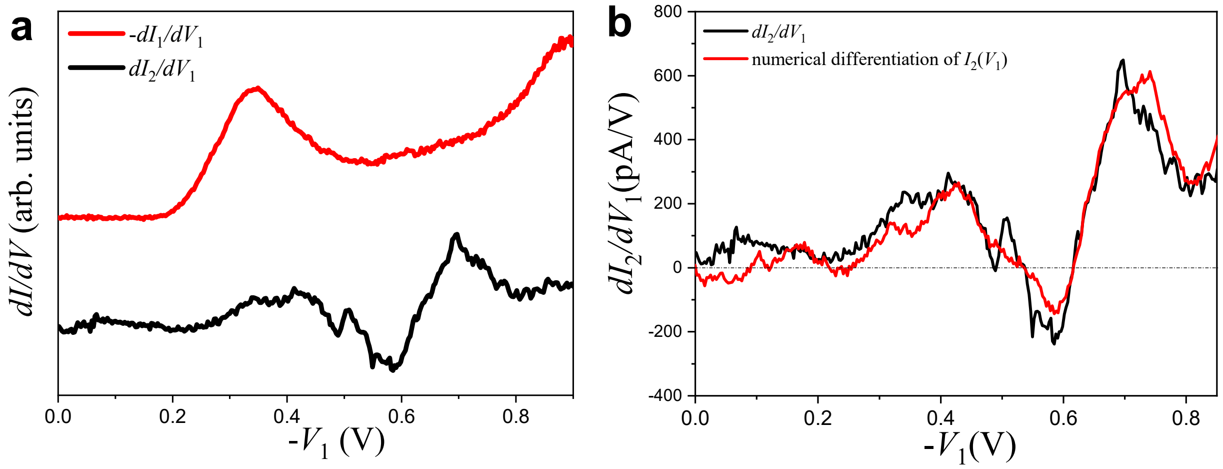

Supplementary Figure 5. **Transconductance from DC I_2 current signal.** (**a**) $-dI_1/dV_1$ (red) and transconductance dI_2/dV_1 (black) 2P-STS data as a function of tip1 voltage obtained simultaneously by application of two lock-in amplifiers for a probe-to-probe distance of about 30 nm from the same set of experimental data as in **Supplementary Figure 3**. Note that dI_2/dV_1 shows resonances at about 0.35 eV and 0.7 eV. (**b**) Comparison between numerical and electronic differentiation of $I_2(V_1)$ signal showing their direct correspondence. The scale in **b** is obtained from numerical differentiation. Note reproduction of negative differential transconductance regime between peaks, which was observed in some cases (see also **Supplementary Figure 3** and Kolmer *et al.*[1]).

Supplementary Note 5: Calculation details

Within the SIESTA package[5,6] the valence-electron wave functions are expanded using a linear combination of numerical atomic-orbitals as a basis set and the core electrons are replaced by norm-conserving Troullier-Martins pseudopotentials[7]. After comparing the electronic band structure of bulk Ge obtained using different parameters, we chose for our simulations the combination of local density approximation (LDA) exchange and correlation (xc) functional with CA parametrization[8,9] and non-relativistic pseudopotentials, together with a double-ζ non-polarized (DZ) basis set since it gives a reasonable description of the electronic structure, while keeping the simulation not too computationally expensive (**Supplementary Fig-**

ure 6). The extension of the orbital radii was defined using a 100 meV energy shift[6].

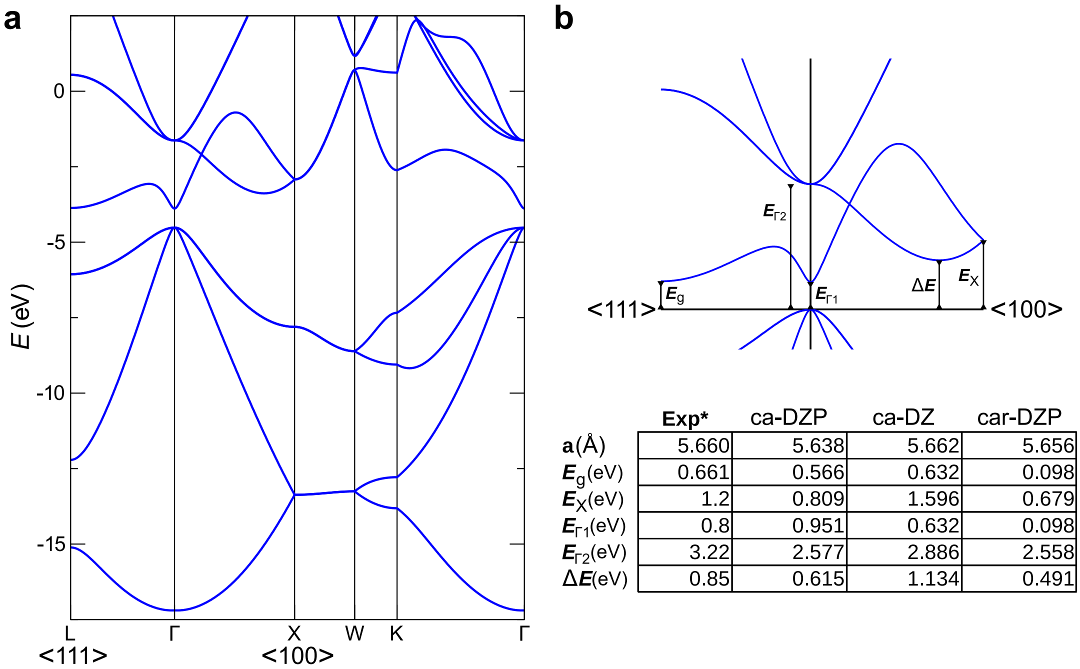

	**Exp***	ca-DZP	ca-DZ	car-DZP
**a**(Å)	5.660	5.638	5.662	5.656
$\boldsymbol{E}_g$(eV)	0.661	0.566	0.632	0.098
$\boldsymbol{E}_X$(eV)	1.2	0.809	1.596	0.679
$\boldsymbol{E}_{\Gamma 1}$(eV)	0.8	0.951	0.632	0.098
$\boldsymbol{E}_{\Gamma 2}$(eV)	3.22	2.577	2.886	2.558
$\Delta\boldsymbol{E}$(eV)	0.85	0.615	1.134	0.491

Supplementary Figure 6. **Band structure of bulk germanium.** (**a**) Ge band structure obtained with local density approximation (LDA) exchange-correlation functional (CA parametrization[8,9]), non-relativistic pseudopotentials and a double-ζ non-polarized (DZ) basis set. (**b**) Comparison of the lattice parameter and characteristic energy differences between calculations and experiments[10].

The fineness of the real space grid was defined using a 250 Ry energy cutoff. Self-consistency was considered to be achieved when the changes of the density matrix elements were less than 10^{-5} as well as lower than 10^{-4} eV for the Hamiltonian matrix elements. The smearing of the electronic occupations was defined by an electronic temperature of 300 K with a Fermi-Dirac distribution. The band structure of the Ge(001)-c(4×2) reconstruction shown in **Fig. 1** of the main text was obtained using a slab containing twelve Ge layers (saturated with hydrogen), consistent with that used later for the transport simulations in 3 and 4-terminal set ups. The k-sampling contained 5×10 k-points.

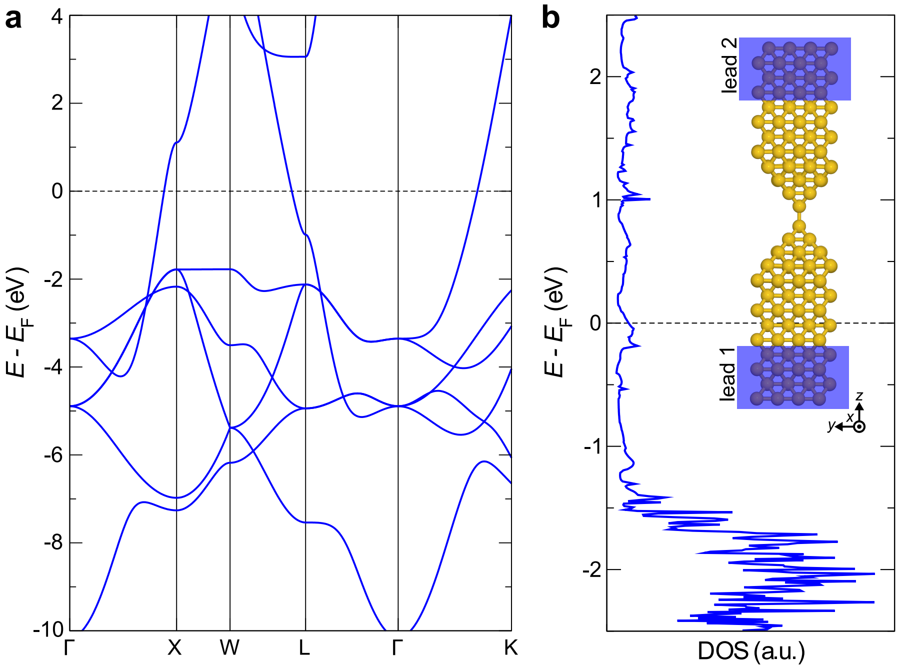

Supplementary Figure 7. **Electronic structure of gold bulk and tip.** (**a**) Band structure of bulk Au obtained with the local density approximation (LDA) functional (CA parametrization[8,9]), scalar-relativistic pseudopotentials and a double-ζ polarized (DZP) basis set for s-orbitals and single-ζ non-polarized (SZ) for d-orbitals (DZP-SZd), obtained with a 20x20x20 k-point sampling of the Brillouin zone. (**b**) Density of states at the scattering (central) region of a gold tip-to-tip system. The inset shows a representation of the gold tip-to-tip system built to verify the transport properties of the Au tip model.

For modeling the metallic tips we used a gold rod with a sharp tip that terminates with a single atom. The Au(100) crystalline direction was taken to define the axis of the corresponding gold rod, which is expected to have a smooth local density of states (DOS) at the tip apex around the Fermi level. Although a tip built from Au(111) would result in a sharper apex, it would not be an ideal choice since it has been reported that such a geometry exhibits resonant states at the Fermi energy, localized at the tip and quite decoupled from extended bulk states[11].

We found that a good description of the bulk Au band structure (especially in the region around the Fermi level) is achieved with CA-LDA xc

functional, double-ζ polarized (DZP) basis set for s-orbitals and single-ζ non-polarized (SZ) basis for d-orbitals (i.e. DZP-SZd), plus a pseudopotential with scalar relativistic corrections. The band structure for bulk Au obtained with these parameters and a 20x20x20 k-point sampling of the Brillouin zone is shown in **Supplementary Figure 7a** and presents a good agreement with reference values[12]. Given the strictly confined nature of the atomic orbitals used in SIESTA, a diffusive s-orbital (radius of 9.0 Bohr) was included in the description of the apex gold atom, in order to improve the description of the tunneling current between the tip and the Ge(001) surface. For all other atoms, the extension of the orbital radii was defined using a 100 meV energy shift[6].

In order to check the consistency of the model we set up a "tip-to-tip" two-terminal device (see inset in **Supplementary Figure 7b**) and calculated the DOS and transmission probabilities via non-equilibrium Green's functions (NEGF) with the TranSIESTA module[13,14]. As shown in **Supplementary Figure 7b**, this system presents a rather smooth non-zero DOS within a relatively large energy range around the Fermi level. As a result, a nonzero transmission probability ($T(E_{\mathrm{F}}) = 1.4$) is observed at the Fermi level. Given the featureless DOS of the gold tips close to E_{F} we can expect that the peaks found in the calculated transmissions presented below are related to the Ge(001) surface band structure.

In order to explore possible changes of the surface geometry due to the proximity of the metallic tip we built up the simulation cell represented in **Supplementary Figure 8**, which comprises a total of 2462 atoms (18221 atomic orbitals), and allowed the Ge atoms closer to the tip to fully relax until the forces were lower than 0.1 eV/Å (highlighted Ge atoms

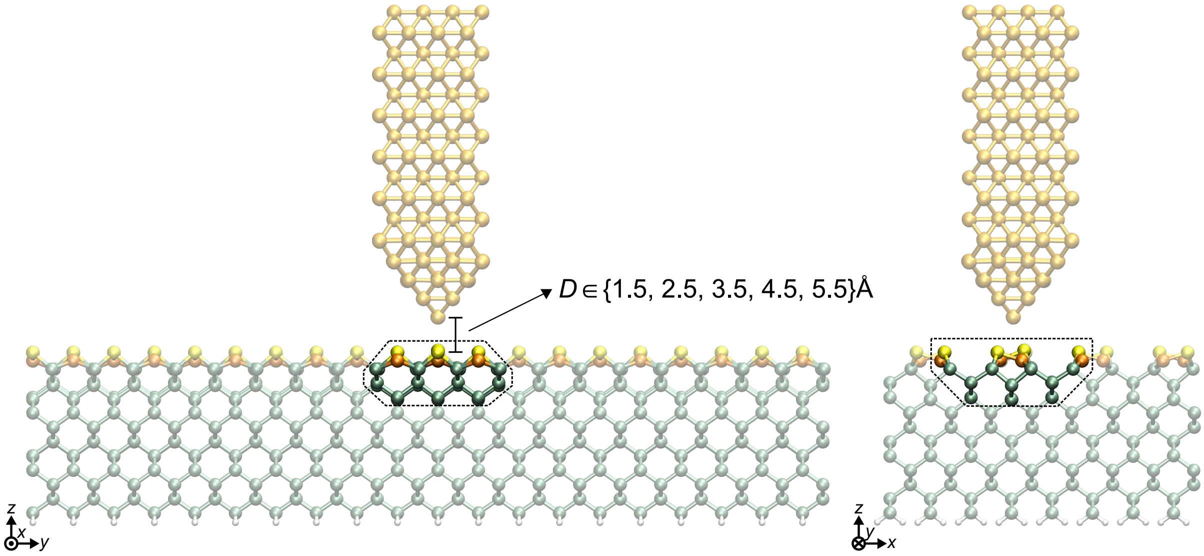

Supplementary Figure 8. **Periodic calculation setup of a Ge(100)-c(4×2) slab and the Au tip model.** The slab comprises four dimer rows of the Ge(100)-c(4×2) reconstruction along the x-direction (perpendicular to the Ge-dimer wires). Ge atoms forming the rows are colored in yellow and orange (all the others are colored in green). The highlighted Ge atoms closer to the metallic tip were allowed to fully relax at five different tip-to-surface distances (1.5, 2.5, 3.5, 4.5, and 5.5 Å).

on **Supplementary Figure 8**), for five different tip-to-surface distances (viz. 1.5, 2.5, 3.5, 4.5, and 5.5 Å). As one would expect, the changes were significantly larger the closer the tip is to the surface, and basically no modification in the geometry happened at a surface-tip apex distance of 5.5 Å.

The same simulation cell described above (**Supplementary Figure 8**) was used to define the transport setup depicted in **Supplementary Figure 9** with a single metallic tip addressing the Ge surface. It comprises three electrodes (blue boxes), two of them at the terminations of the Ge slab (left and right) and another one for the Au tip model. The unit cells defining the Ge electrodes contain 224 atoms each and their Hamiltonians were obtained from a periodic calculation with a 3x20 k-point sampling,

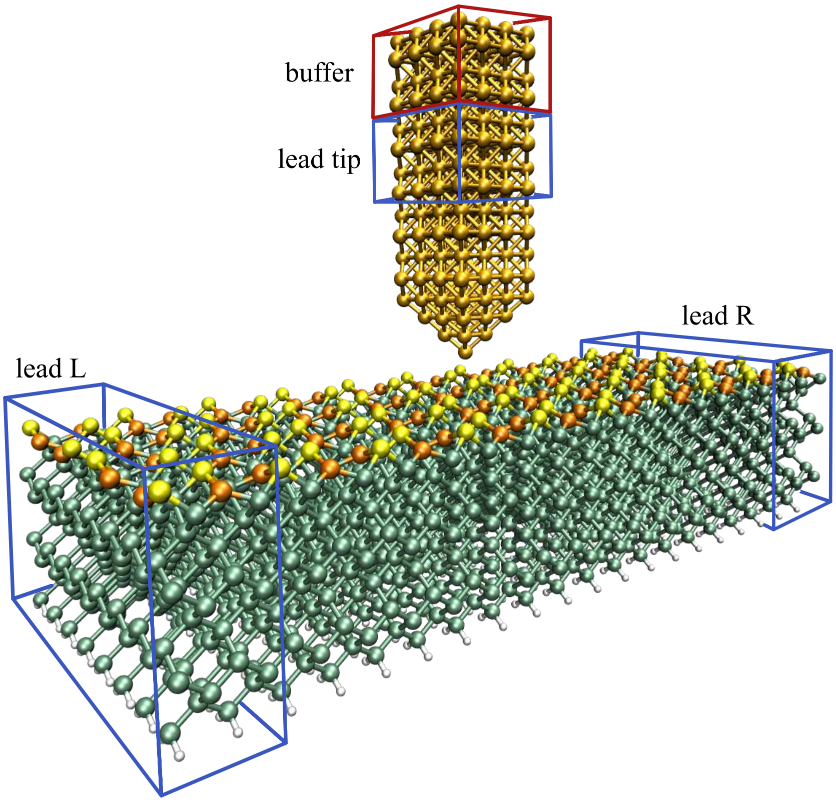

Supplementary Figure 9. **Representation of a 3-terminal setup for transport calculations.** Two electrodes are defined at the Ge slab terminations and a third electrode is defined for the Au tip, where a region with buffer atoms has been used to improve the convergence at the interface between electrode and scattering region. Ge atoms are colored in green, except for those forming the buckling wires which are colored in yellow and orange.

being the larger amount along the transport direction. The same k-point sampling of the Brillouin zone was used for the Au electrode, which was defined by a unit cell containing 64 atoms (4 layers in the metallic rod). Since for the later case there are no periodic boundaries in its semi-infinite direction, we used buffer atoms (red box) to help the convergence of the density matrix at the interface between the scattering region and the metallic electrode, i.e., avoiding spurious effects due to the artificial surface at the top of the gold tip. Basically these extra atoms are used only on the initial calculation in order to provide a better initial guess to the Hamiltonian in the Au electrode region, but they are not considered further in

the transport calculation part. Besides, we considered ∼30 Å distance of empty space between the buffer region and the periodic Ge slab replica. In all our transport simulations, to obtain the Hamiltonian of the full open system and to compute the transmission probabilities, 3 k-points were considered along the transverse direction, i.e., perpendicular to the electrodes semi-infinite directions. For the Ge electrodes the imaginary part of the surface Green's function was set to $\eta = 10^{-5}$ eV, while for the Au lead we adopted $\eta = 0.02$ eV.

Before proceeding with the transport simulations, an important issue must be addressed, namely the level alignment between the metallic and semiconducting leads and the scattering region. The Au lead has a well-defined Fermi level as well as the central scattering region where, due to the large amount of Au atoms defining the tip in contact with Ge surface, the Fermi level in our initial periodic SIESTA calculation is also expected to be well-defined and to be physically meaningful. However, there is no clear way to define the electrochemical potential (Fermi level) in the semiconducting Ge electrodes, where any position of the Fermi level within the band gap is physically reasonable.

To solve this problem we devised the following method to align the Ge leads with the other simulation parts. First we perform a periodic calculation of the scattering region for a given tip-to-surface distance from where we take the DOS projected onto the farthermost Ge slab atoms (rectangular box in **Supplementary Figure 10a**). A similar projection is done for the Ge lead calculation (**Supplementary Figure 10a** in the right). By plotting both PDOS on top of each other one can then directly read the required energy shift to align the levels. The continuity of the

potential at the interface between the scattering region and the leads must be reflected in the possibility to superimpose the PDOS computed to the right (inside the electrode) and to the left (inside the scattering region) of that artificial interface.

In **Supplementary Figure 10** this procedure is illustrated for the case of 3.5 Å tip-to-surface distance. As shown in **Supplementary Figure 10b**, by superimposing the PDOS computed at the boundary of the scattering region (blue curve) with that computed at the Ge slab lead (green curve), one realizes that the correct level alignment is achieved by applying a 83 meV rigid shift to the lead energy levels (red curve). The effect of the described procedure in a calculation with open boundaries is presented in **Supplementary Figure 10c**, where the results from a non-aligned calculation were included for comparison. Significant changes are found, specially in the energy window dominated by the empty surface states coming from the dimer wires, i.e. [-4.7,-3.8] eV. After alignment, the DOS shows clearly defined van Hove singularities that follow closely the onset of the surface bands of Ge(001). The left-to-tip transmission (right of **Supplementary Figure 10c**) is considerably modified with the level alignment, particularly at the energies corresponding to the Ge slab band edges. The shape and width of the transmission features associated with the surface bands is in good correspondence with the pristine system and we only find a reduction due to the scattering induced by the proximity of the tip apex.

This energy level alignment method described above was applied to all five different tip-to-surface distances, namely 1.5, 2.5, 3.5, 4.5, and 5.5 Å (inset in **Supplementary Figure 11a**). In **Supplementary Figure 11b**

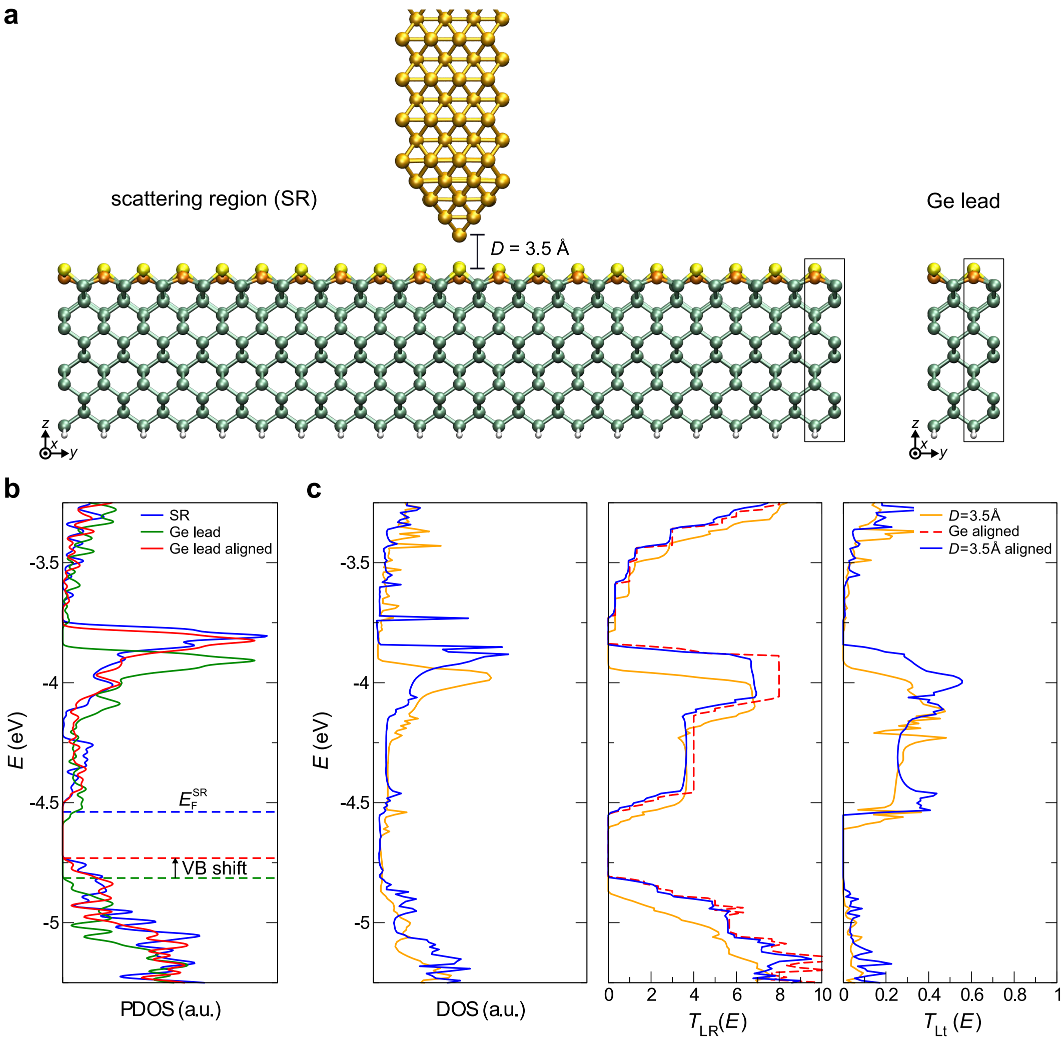

Supplementary Figure 10. **Energy alignment procedure for transport calculations.** (**a**) Representation of the scattering region (SR) for a simulation with a 3.5 Å tip-to-surface distance (left) and the unit cell of the Ge electrode (right). Ge atoms forming the dimer rows are colored in yellow and orange (the others are colored in green). The density of states (DOS) projected on the atoms inside each of the rectangular boxes was chosen to perform the alignment procedure. (**b**) Projected density of states (PDOS) on the farthermost Ge slab atoms (rectangular boxes in SR in panel a). The PDOS in the Ge lead, as initially computed, is shown in green. In order to get a good superposition of the PDOS computed in the scattering region (blue) and for the lead (green), it is necessary to apply a shift of 83 meV (red curve). (**c**) Comparison between aligned and misaligned open boundaries calculations: DOS obtained from Green's functions (left), transmission functions from left-to-right (center) and left-to-tip (right). Orange curves show the results using the Ge lead referred to the arbitrary Fermi level as it comes from the initial periodic calculation, blue curves present the results after applying the procedure described above. Red dashed line shows the expected transmission for a pristine Ge(001) surface.

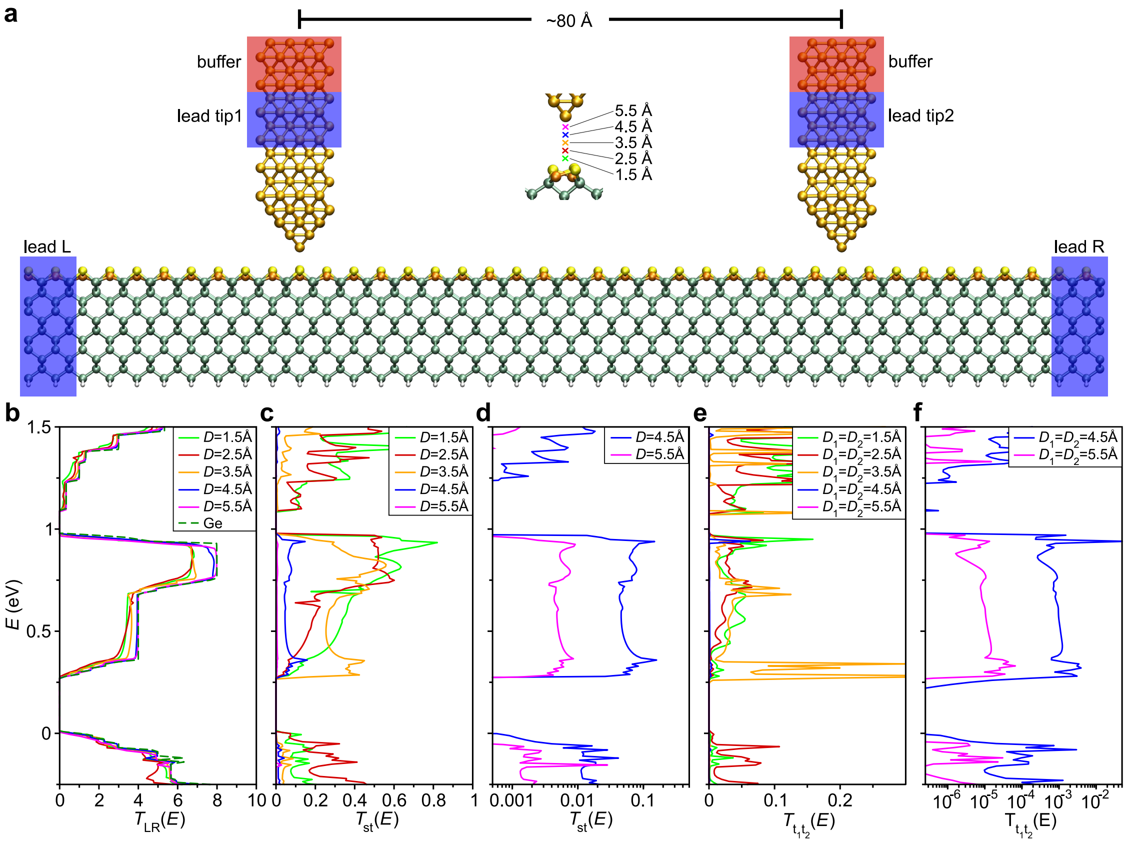

Supplementary Figure 11. **Simulation of the 2-probe experiment.** (**a**) 4-terminal setup for simulating the transport properties of the 2-probe experiment. Ge atoms are colored in green, except for those forming the buckling wires which are colored in yellow and orange. The electrodes are highlighted by blue boxes, two of them located at the Ge slab terminations (namely left and right leads) and the other two placed at each Au model tip (tip1 and tip2 leads). As in the single tip case **Supplementary Figure 9**, two regions of buffer atoms were included in each tip termination in order to improve the convergence at the electrode/device interface. In this setup the tip apexes are aligned on the same Ge dimer row (our Ge slab is defined by 4 Ge dimer rows in a 4x2 reconstruction) and positioned 80 Å apart. Inset shows the five different tip-to-surface distances were considered, namely $D \in 1.5, 2.5, 3.5, 4.5, 5.5$ Å. (**b**) Left-to-right transmission functions with a single tip, i.e. 3-terminal setup (**Supplementary Figure 9**). The transmission through an isolated Ge slab is included in dashed green as a reference. (**c**) Surface-to-tip transmissions, i.e., sum of the transmission probabilities from each of the Ge electrodes to the metallic tip, for a single tip setup. (**d**) Same as **c** for the two largest tip-to-surface distances, in log-scale. (**e**) Tip-to-tip transmission functions obtained with the full 4-terminal setup. (**f**) Same as **e**, in log-scale. All transmissions were evaluated at zero bias, and all curves were averaged over the transversal k-points and aligned with respect to the Ge slab valence band edge.

we present the transmission function from left-to-right Ge leads in a single-tip setup for the different considered tip-to-surface distances. One can see the increase of the scattering as the tip approaches the Ge surface. The surface-to-tip transmissions for the same single-tip configuration are shown in **Supplementary Figure 11c-d**, where an inverse relation is observed, i.e., an increase of the transmission the closer is the tip.

Finally, the two-probe experiment was simulated with the 4-terminal setup shown in **Supplementary Figure 11a**, which is defined by two Ge electrodes on each slab termination and two Au tips. The whole system is defined by 4924 atoms, which corresponds to 36442 atomic orbitals, in a supercell of dimensions 32.03x160.15x80.0 Å^3. The tip-to-tip transmission of the full 4-terminal setup is presented in **Supplementary Figure 11e-f**, where both tips are positioned at the same height. One clearly see that the resonance at 0.7 eV decreases significantly for tip-to-surface distances $\geq$ 4.5 Å. Note also that the appearance and precise energy position (within $\sim$0.05 eV) of the CBE+1 resonance also depends on the exact tip-to-sample distance. The lead-resolved DOS presented at **Fig. 5c-d** are calculated from the diagonal elements of the lead spectral function[14], which for lead j and energy E is defined as $A_j(E) = G^r \Gamma_j G^{r\dagger}$, where $G^r(E)$ is the scattering region retarded Green's function and $\Gamma_j = i\left(\Sigma_j^r - \Sigma_j^r\dagger\right)$ is the coupling matrix, with Σ_j^r the lead j retarded self-energy.

Supplementary Note 6: Step-edge reflection experiment and simulations

The simulation setup of a two-terminal model of the step-edge with dimensions comparable to the experimentally probed area is presented in **Supplementary Figure 12a**. As in the experiment (**Supplementary Figure 12b**), the dimer wires in one of the terraces are perpendicular to those in the following terrace, with one atomic layer difference in height (the experimental morphology analysis in **Supplementary Figure 12c** indicates a monoatomic step). Given the orientation of the dimer wires with respect to the step edge, it is not possible to build a simulation cell of reasonable dimensions with periodic boundary conditions to represent the system. It would be necessary to include two steps and advisable to increase substantially the size of the cell to avoid possible spurious effects due to the scattering in this second step. For this reason we used a finite system along the transport direction, increased by the addition of buffer atoms (red boxes in **Supplementary Figure 12a**) in order to get a better initial guess of the electronic Hamiltonian and to help the convergence of the density matrix at the interface between scattering region and electrodes. In total, the setup comprises 3236 atoms (22888 orbitals) in a 16.01x244.2x50.0 Å^3 cell.

Supplementary Figure 13a shows the square modulus of the right-electrode eigenchannels at specific energies for Γ_{perp}, i.e. zero crystalline momentum in the direction parallel to the step-edge. The eigenchannels are defined as linear combinations of scattering states originated in a particular electrode that diagonalize the transmission matrix through

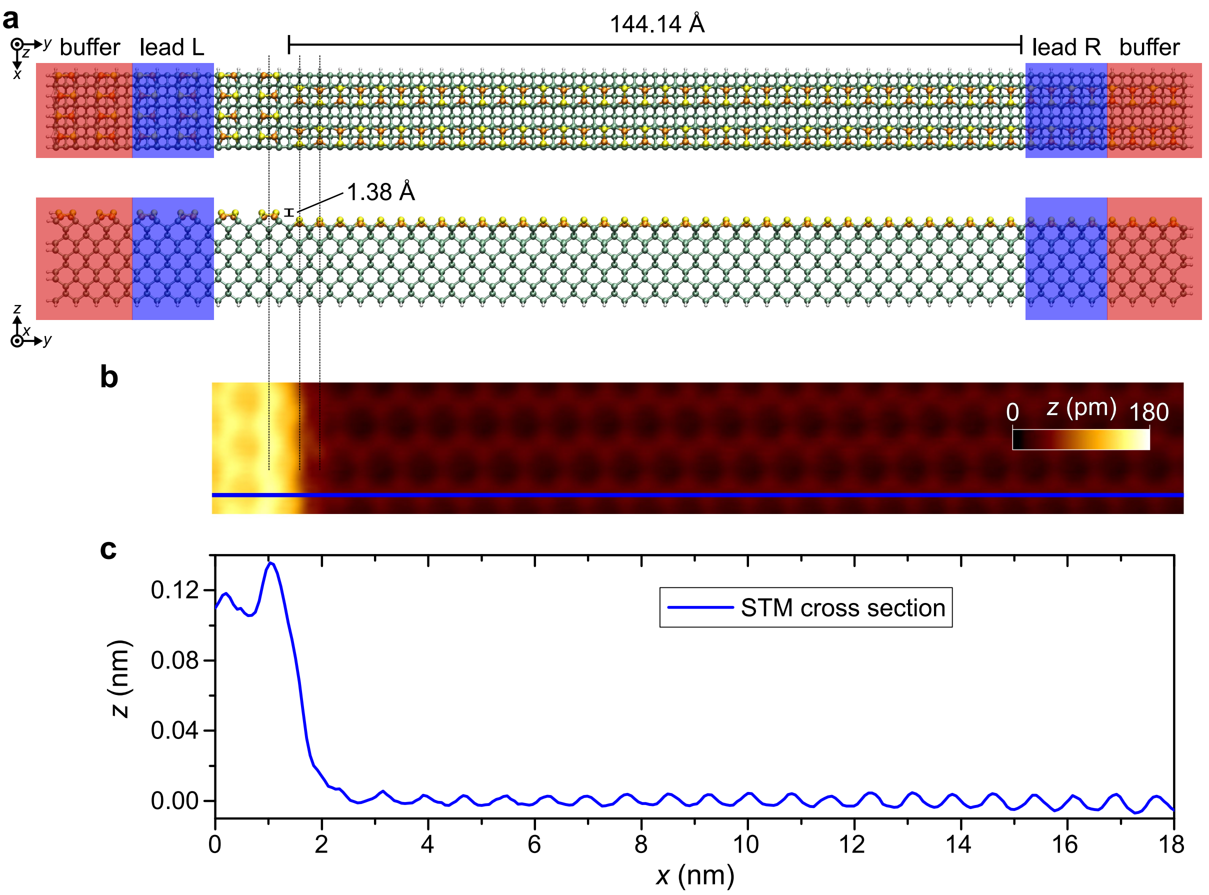

Supplementary Figure 12. **Step edge calculation setup and experimental morphology.** (**a**) Representation of the step edge setup used in the transport calculations. Ge buckled dimers are colored in yellow and orange (all the other Ge atoms are colored in green). The step height corresponds to one atomic layer (1.38 Å). The dimer wires (yellow and orange colored Ge atoms highlight the buckled wire) in the higher and lower terraces are oriented perpendicularly to each other. A very large device region of 144.14 Å has been defined in the lower terrace, such that it can contain several oscillations of the standing-waves formed by the scattering at the step edge. Two electrodes are included, one in each termination (blue boxes), and additional layers of buffer atoms were used (red boxes) to improve the convergence at the interface between electrode and scattering region. (**b**) Filled-state scanning tunneling microscopy (STM) image (100 pA, -0.5 V) of the atomically perfect surface area near the step-edge, with the same scale as the model in **a**. (**c**) STM morphology characterization in the direction perpendicular to the step-edge, showing a step height of ∼1 Å, i.e., approximately one atomic layer.

the system[15], and they were calculated here using the Inelastica package[16] (`https://github.com/tfrederiksen/inelastica.`) from the results of the TranSIESTA calculation. Since an eigenchannel is in principle a prop-

agating wave, it can present a non-zero imaginary part and, therefore, we have chosen to present here only their moduli. We might interpret them simply as giving the probability distribution to find electrons that are injected from the right electrode at a given energy and propagating towards the device region, and given the chosen energy normalization their amplitudes correspond to a local DOS[15]. Most of the eigenchannels are almost completely reflected at the step-edge, thus, creating well-defined standing-wave patterns. Only a few of them propagate with a given transmission probability to the upper terrace at the other side of the step-edge. Note that the number of eigenchannels at a given energy is equal to the states available in the right lead at that energy. The red dashed lines over the band structure in **Supplementary Figure 13b** indicate the energies at which the eigenchannels are evaluated. When the line crosses a single band only one eigenchannel is found. At a higher energy two bands are available and, accordingly, two eigenchannels are found, and so on. Nevertheless, we were able to select the same state by identifying the wave function symmetries and, accordingly in **Supplementary Figure 13a**, the wave functions marked with a blue star correspond to the same eigenchannel coming from the lowest empty surface band in the right-lead.

The calculated interference patterns exhibit a remarkable agreement with the experiment (**Figs. 2b-c** in the main text). Starting from E = 0.29 eV (defined with respect to the valence band edge in the slab calculation), where three maxima are observed in the device region similarly to the measurement at 0.30 eV (**Figs. 2b-c**, main text), a direct correspondence between the calculated wave functions and the dI/dV maps can be delineated for higher energies. In the experiment it is harder to select the

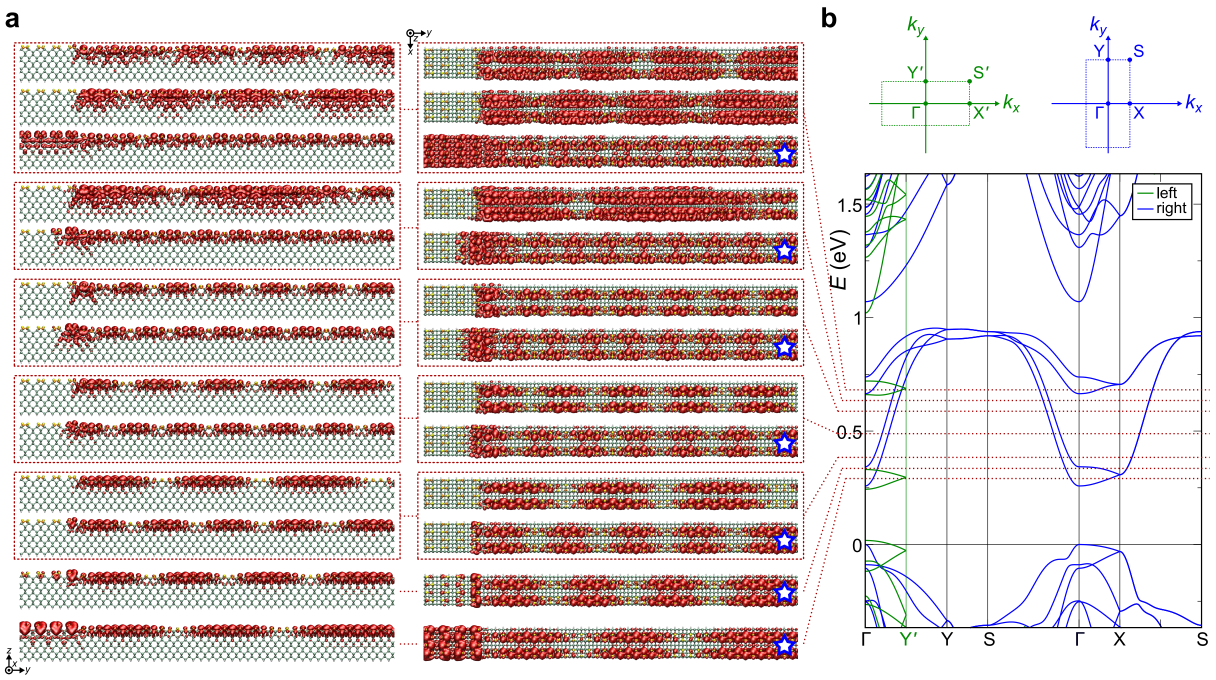

Supplementary Figure 13. **Surface states scattered at a monoatomic step edge.** (**a**) Eigenchannel scattering states incoming from the right lead, calculated at Γ_{perp} and for the energies indicated by the dashed red lines in the band structure in panel **b**. Red boxes group the multiple eigenchannels calculated at a given energy. Ge buckled dimers are colored in yellow and orange to distinguish them from the other Ge atoms, which are colored in green). (**b**) Band structures of the left (green) and right (blue) electrodes. The corresponding Brillouin zones are shown above.

signal from a single conducting state though and, therefore, at higher energies the measurements are associated to a convolution of multiple wave functions.

The side view of the calculated wave functions in **Supplementary Figure 13a** also indicates that the interference patterns are highly localized at the surface, as expected from the comparison between the slab and bulk Ge band structures in **Fig. 1** from the main text showing that the dispersive bands in the energy range from ∼0.3 to ∼0.9 eV have a clear surface character. Additionally, the simulation shows that for energies from 0.36 to 0.66 eV the corresponding wave functions are reflected with no forward

scattering through the step-edge. However, for low energies below 0.36 eV and energies exceeding 0.66 eV, the eigenchannels demonstrate a non-zero probability of overcoming the step-edge barrier. The main reason behind this behavior is explained in **Supplementary Figure 13b**. There, we can see that around those energies the band structure (for Γ_{perp}) of the right electrode (Ge dimer rows parallel to the transport direction, blue bands) becomes resonant with the bands of the left electrode (Ge dimer rows perpendicular to the transport direction, green bands). This is a necessary condition to have transport across the step in our elastic transport calculation. In addition, we can expect that the larger two-dimensional character of the band structure at those energies (see **Supplementary Note 9**, **Supplementary Figure 20**) will also contribute to facilitate the transmission, making the barrier more transparent. Such observation may explain the experimentally observed general weakening of LDOS modulation signal for energies above 0.7 eV (**Fig. 3d** in the main text). Finally, for energies above 0.9 eV we expect strong electronic coupling with the bulk conduction band edge, which contributes to a complete loss of the observed LDOS oscillations in the experiment.

The complete reflection of quasi-particles for the energy range of interest allows to use the back-scattering process to experimentally reconstruct the band structure of the Ge dimer wires[2,17,18]. Reconstruction of Ge(001)-c(4×2) band structure along Ge dimer rows is done by introducing a scattering vector $\mathbf{q}$ with the length of the double length of the corresponding quasi-particle wave function vector $\mathbf{k}$ according to the following formula $|\mathbf{q}| = |\mathbf{k}_{\mathrm{i}} - \mathbf{k}_{\mathrm{r}}| = 2k$, where $\mathbf{k}_{\mathrm{i}}$ and $\mathbf{k}_{\mathrm{r}}$ are the incident and reflected quasi-particles wave vectors, respectively. The experimental data in **Fig. 3d**

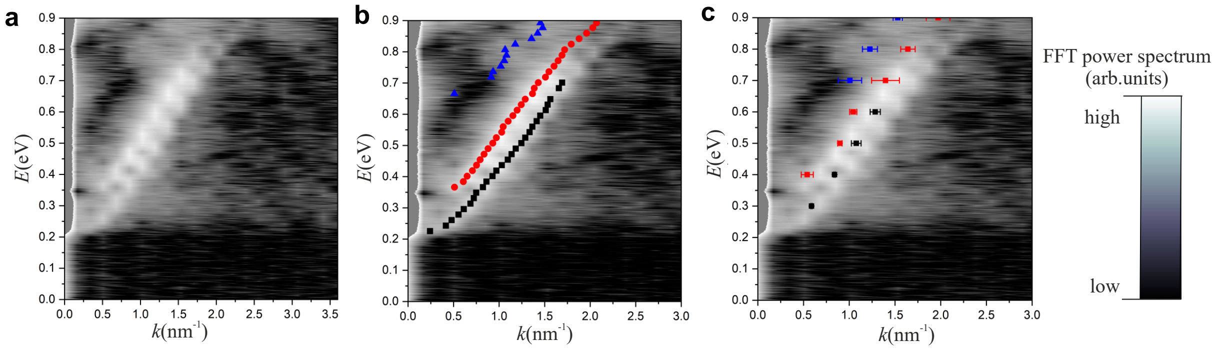

Supplementary Figure 14. **Fourier transform (FT) scanning tunneling spectroscopy. (a)** Raw one-dimensional FT of the data presented in **Fig. 3d**. **(b)** The same FT data with superimposed square, triangular and circle $E(k)$ points obtained by fitting procedure applied to constant energy cross-sections of dI/dV data from **Fig. 3d** (see examples in **Supplementary Figure 15a-c**). Note that our procedure could only capture two wave vectors. **(c)** The same FT data with superimposed $E(k)$ points obtained by fitting procedure applied to constant current dI/dV maps from **Fig. 3b** (see examples in **Supplementary Figure 15d-f**). Error bars correspond to standard deviation obtained from 25 dI/dV line cross-sections. Note good matching between both type of point data and FT background.

from the main text comprise spectroscopy points acquired for 512 energy (voltage bias) points from 0 to 0.9 eV at 47 positions equally spaced by 0.4 nm on the same Ge dimer row. The one dimensional Fourier transform (power spectrum) of these data presented in **Fig. 3e** as a background (right part) was done with the use of WSXM software[19]. In a raw form it is shown in **Supplementary Figure 14a**. The points describing the dispersion of the surface bands in **Fig. 3e** and **Supplementary Figure 14b** were obtained by a separate analysis of the spatial pattern for each energy value. First, to reduce noise, the signal from ten adjacent energy points were averaged out. This led to the energy resolution of about 0.018 eV. Then, for every line we fitted a trigonometric function multiplied by a dying-off exponential function:

$$f(x) = A\exp(-x/L)(\cos(2k_1x - \phi_1) + B\cos(2k_2x - \phi_2)) + C\,, \qquad (1)$$

where A, B, C, L, ϕ_1, ϕ_2, k_1 and k_2 are parameters. In this formula we generally assume two dominant wave vectors. We also assume here that the interference patterns are independently formed for each band. For energies below ~0.35 eV we used only single cosine function (B=0) following Nakatsuji *et al.*[2], Sagisaka and Fujita[17]. Similar procedure could be performed for cross-sections of constant current dI/dV maps presented in **Fig. 3b**. In this case the spatial resolution of data is increased to 0.07 nm but the additional term related to unit cell modulation must be also included. It has $\cos(k_3x - \phi_3)$ form, where k_3 is in the order of 8/nm (~0.8 nm modulation). $E(k)$ points resulting from these fits are presented in **Supplementary Figure 14c**. **Supplementary Figure 15** presents the examples of raw dI/dV data for chosen constant energy cross-sections **(a-c)** and dI/dV constant current maps cross-sections **(d-f)** with corresponding fits.

The parameters k_1 and k_2 obtained by fitting allow to draw dispersion relations between the quasi-momentum and the energy $E(k)$ for three bands (#1, #2 and #3,#4). The corresponding effective masses were obtained by parabolic fits to these three $E(k)$ relations for k values up to 1.5 nm^{-1}. The experimental values of 0.18±0.04 m_e (band #1 and band #2) and 0.35±0.1 m_e (band #3,#4) are in agreement with the calculated band structure, from which the corresponding effective masses in #1-4 bands are 0.19, 0.20, 0.38 and 0.41 m_e, respectively.

Figs. 2c-d from the main text show a clear dependence of the coherence length as a function of the quasi-particle energy. The largest coherence is observed for energies close to the lower edge of the conduction surface band (CBE resonance) at about 0.4 eV, for which the interference patterns

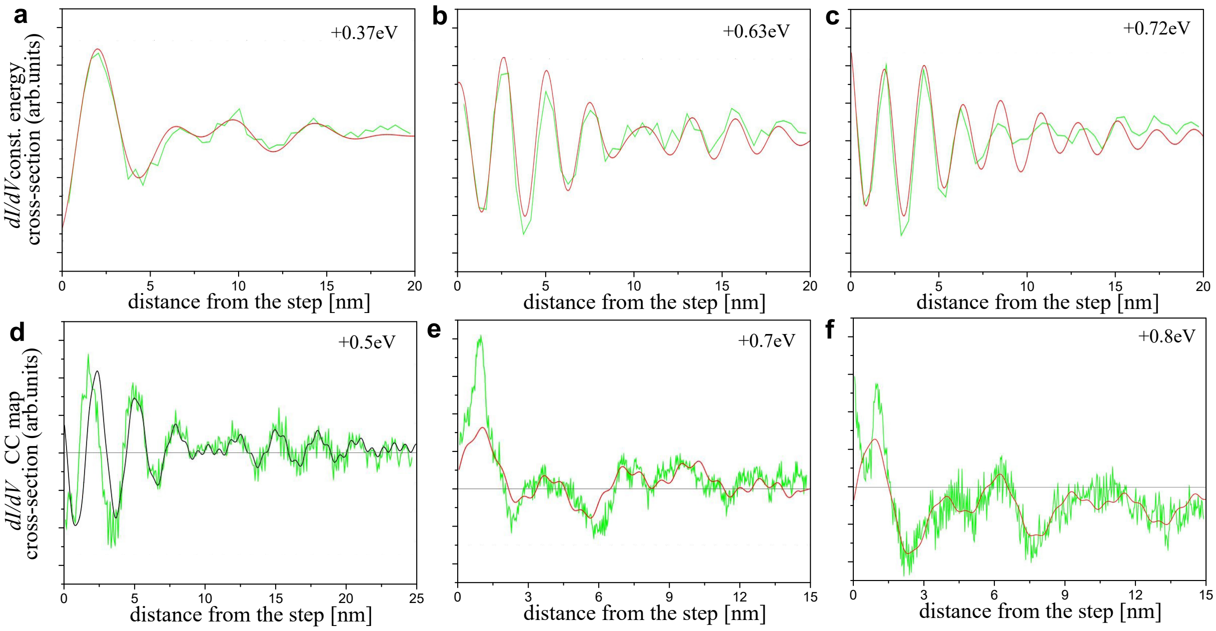

Supplementary Figure 15. **Scanning tunnelling spectroscopy** dI/dV **cross-sections near the step-edge. (a-c)** Examples of dI/dV constant energy cross-sections from data presented in **Fig. 3d** (green curves) with corresponding fits (red curves). **(d-f)** Examples of dI/dV constant current map cross-sections from data presented in **Fig. 3b** (green curves) with corresponding fits (black and red curves). For lower energies the fits are performed for longer distances (24nm, black curve in **d**) than for higher energies (15nm, red curves in **e** and **f**). Note that single frequency could not reproduce the data in any of the presented dI/dV distance dependence.

disappear only for distances of ∼25 nm as described in the main text. Interestingly for energies exceeding 0.7 eV the interference patterns vanish quicker. Here, we argue that the effect is presumably related to more two-dimensional character of corresponding bands above 0.7 eV as shown in our k-resolved DOS calculations in **Supplementary Figure 20**.

Supplementary Note 7: Transmission probabilities with a single Au tip

Here we analyze how the electronic transmission through the surface states from a Ge(100)-c(4×2) slab is affected when addressed by a single Au tip positioned at 3.5 Å from the surface (as in **Supplementary Figure 10**). **Supplementary Table 1** shows the transmission probabilities decomposed on the eigenchannels available at two different energy intervals where the Ge(001)-c(4×2) surface states resides, namely [0.38, 0.68] eV and [0.73, 0.93] eV (given with respect to the valence band). For the first energy interval [0.38, 0.68] eV, where only CBE is present, only one channel exhibits a significant reduction on transmission of about 39%, indicating that such channel should correspond to the dimer row over where the Au tip apex is centered on top (note that there are four dimer rows in the simulated cell). In the second energy interval [0.73, 0.93] eV, where CBE and CBE+1 coexist, one channel suffers again a similar reduction on transmission (∼37%), however another one is almost completely suppressed by 90%. This again suggests that the transmitting channels that present a large reduction are related to the dimer row addressed by the metallic tip, being the channel corresponding to CBE+1 more strongly affected.

Supplementary Note 8: Surface states

Before analyzing in more detail the characteristics of the surface states, let's first clarify the consequence of our choice of a double-sized unit cell in the band structure analysis. **Supplementary Figure 16a** shows in the left a primitive cell of the Ge(001) surface, which for the c(4×2) reconstruction is defined by an hexagonal lattice, and in the right the unit cell used in this work with orthogonal lattice vectors, which is a more natural choice to compare with the relevant directions of the performed experiments.

In **Supplementary Figure 16b** it is presented the band structure of the Ge(001)-c(4×2) slab calculated with the primitive cell along the two paths depicted in red and green at the Brillouin zone from panel **a**. One can note that the combination of these two paths (i.e., folding of the green path into the red) is equivalent to the band structure computed using the orthogonal

eigenchannel	transmission at [0.38, 0.68] eV	transmission at [0.73, 0.93] eV
1	99.86%	99.90%
2	99.75%	99.88%
3	99.60%	99.77%
4	61.29%	99.18%
5	–	98.14%
6	–	94.53%
7	–	63.24%
8	–	10.07%

Supplementary Table 1. **Transmission probabilities per channel.** Transmission eigenvalues from one Ge lead to the other (left-to-right) for a Ge slab addressed with a single Au tip at $D = 3.5$ Å from the surface (as in **Supplementary Figure 10a**), averaged in the energy interval [0.38, 0.68] eV (center) and [0.73, 0.93] eV (right), with respect to the valence band. The transmission are also averaged over transversal k-points.

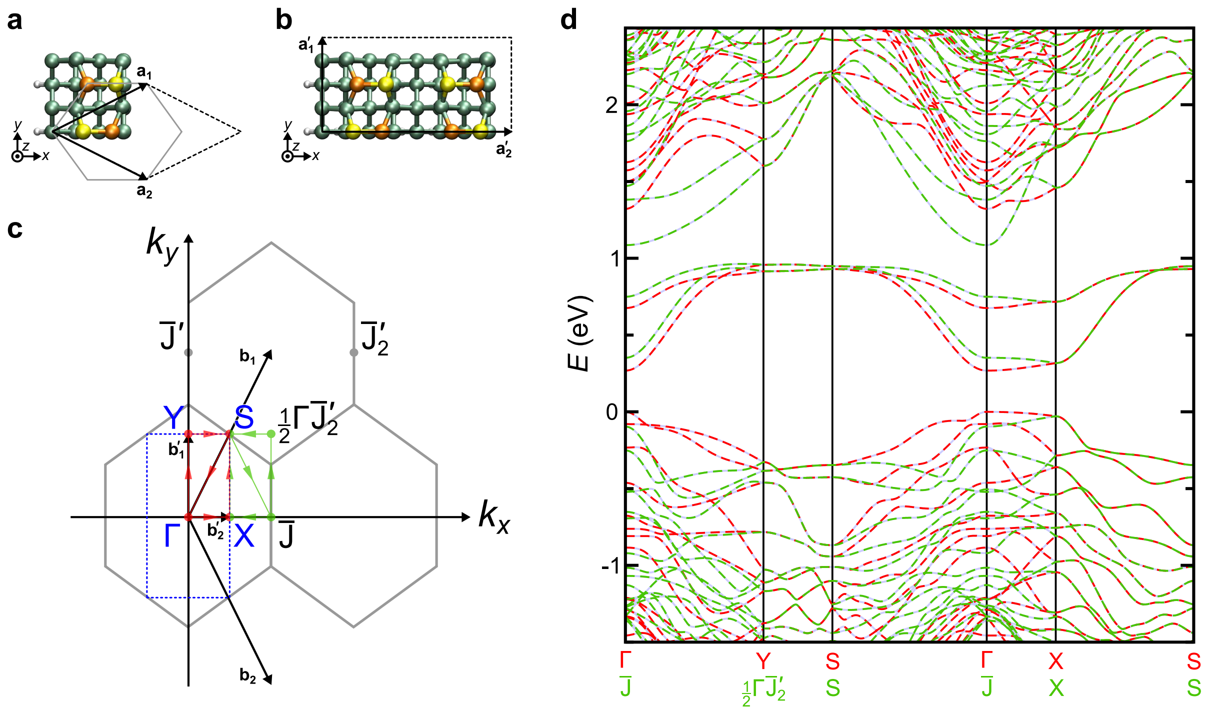

Supplementary Figure 16. **Electronic structure of Ge(001)-c(4×2) slab.** (**a**) Ge(001)-c(4×2) slab primitive cell, whose primitive vectors form an hexagonal lattice. Ge buckled dimers are colored in yellow and orange to distinguish them from the other Ge atoms, which are colored in green). (**b**) A double-sized unit cell chosen in this work, defined by orthogonal lattice vectors. (**c**) The corresponding Brillouin zones, in gray for the hexagonal lattice and in blue for the orthogonal one. (**d**) Band structure calculated using the primitive cell along the red and green paths depicted in the Brillouin zone shown in **c**. This explains clearly the band-folding effects induced by the use of a doubled orthogonal cell.

cell (**Fig. 1a** in the main text and **Supplementary Figure 17b** below), whose corresponding Brillouin zone is shown in blue in panel **a**.

We now proceed with the analysis of the empty surface states of a Ge(001)-c(4×2) slab. **Supplementary Figure 17** shows the band structure calculated with the chosen orthogonal cell and the density of states on all Ge atoms and projected (PDOS) on those Ge atoms belonging to the dimer rows at the surface (also the projection on different atomic orbitals is shown in this latter case). One can note that the DOS in the energy window defined by the red dashed lines is almost completely given by the

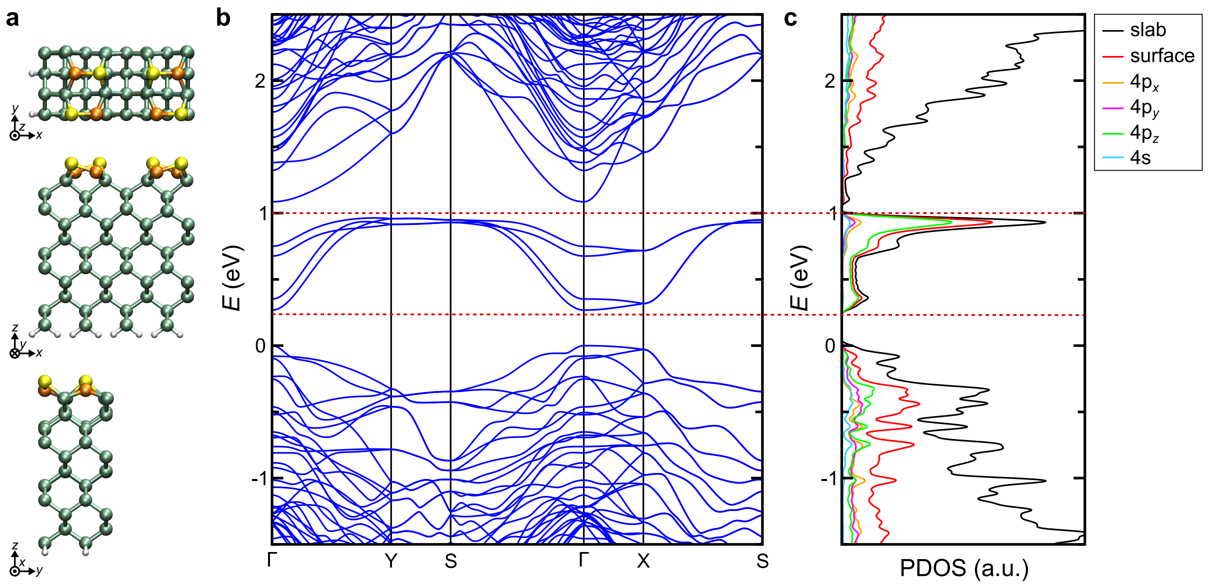

Supplementary Figure 17. **Orbital character of the Ge(001)-c(4×2) surface states.** (**a**) Unit cell of a twelve-layer Ge(001)-c(4×2) slab. Ge atoms forming the buckling rows are highlighted in yellow and orange (other Ge atoms are colored in green). (**b**) Calculated band structure. Red dashed lines indicate the energy window containing the empty surface states. (**c**) Density of states projected on all Ge atoms (black), on Ge atoms at the surface defining the dimer rows (red), and on p_x (orange), p_y (magenta), p_z (green) and s (blue) orbitals of those atoms. All energies are given with respect to the valence band top in the slab. For the PDOS calculation we used a k-point sampling of 5×50 ($k_x \times k_y$).

Ge atoms forming the dimers and that it is dominated by a π character ($4p_z$ orbitals of Ge).

In the Ge(001) surface, the wave function corresponding to the CBE+1 band shows a strong sign oscillation along the dimer wire (**Supplementary Figure 18**). Therefore, given its large p_z character a fast decay rate of the STS signal is also expected over the dimer row. To further support this observation we performed STM simulations using the surface Green-function matching (SGFM) method[20] with an extended Hückel molecular orbital Hamiltonian parameterized by fitting accurate DFT band structures. This methodology was optimized to simulate STM images on Ge(001), where it provides images in excellent agreement with experiment.[21] The simula-

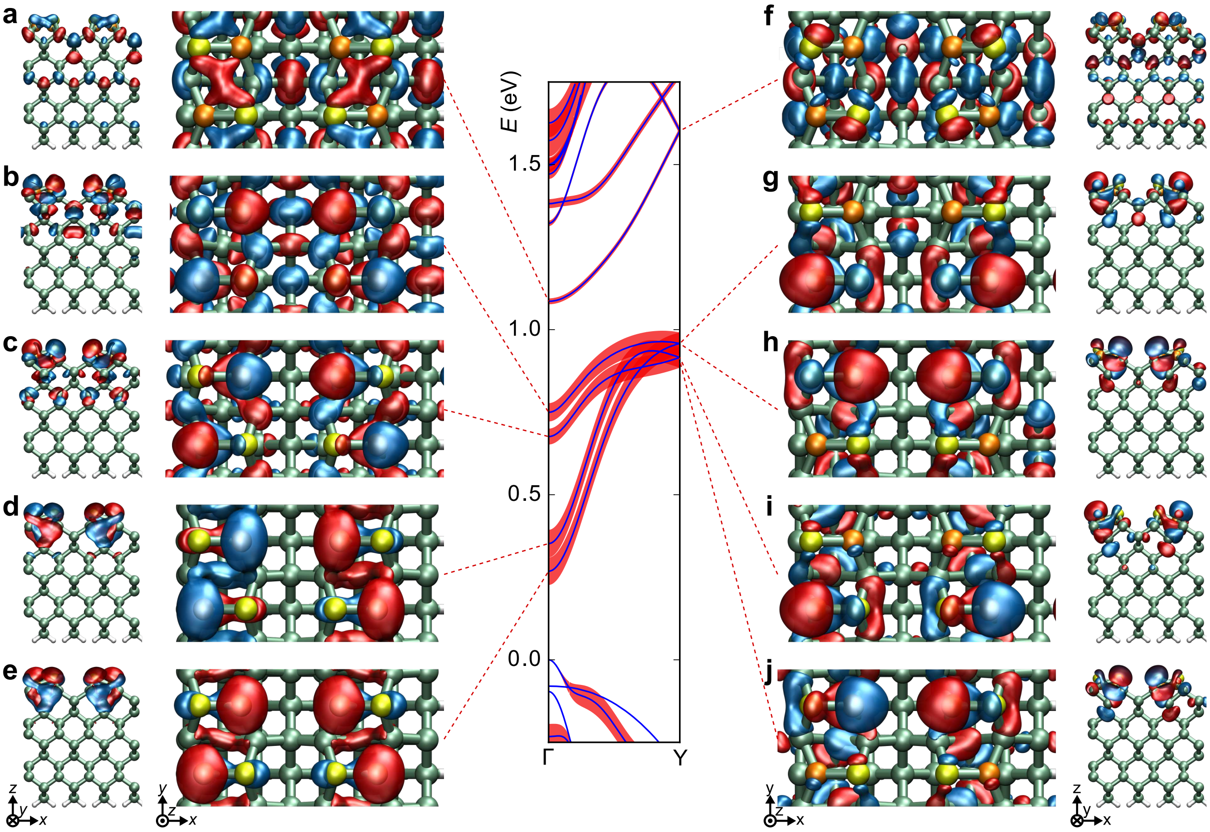

Supplementary Figure 18. **Ge(001)-c(4×2) wave functions.** Real part of the wave functions corresponding to the bulk conduction band (**a** and **f**) and surface states calculated at Γ (**b-e**) and at Y (**g-j**). The band structure is shown in the center, where the size of the red shaded area around a given band represents the amount of p_z character. Red dashed lines indicate the energies where the wave functions were evaluated. Note that the wave functions corresponding to unoccupied bulk Ge (**a** and **f**) show a non-zero amplitude over the entire slab, while those associated to the surface bands are localized in the top Ge layers. The Ge atoms are represented in green except for those forming the buckled dimers which are colored in yellow and orange.

tion setup is shown in **Supplementary Figure 19a**, where the STM tip modeled as a semi-infinite W(111) slab with a Ge-terminated tip address a Ge(001)-c(4x2) surface defined by nine layers on top of a semi-infinite Ge(001) bulk. The calculated transmission functions for different tip-to-surface distances are presented in **Supplementary Figure 19b**, where the peak corresponding to CBE+1 becomes less visible as the tip-to-surface distance is increased. These calculations are in qualitative agreement with

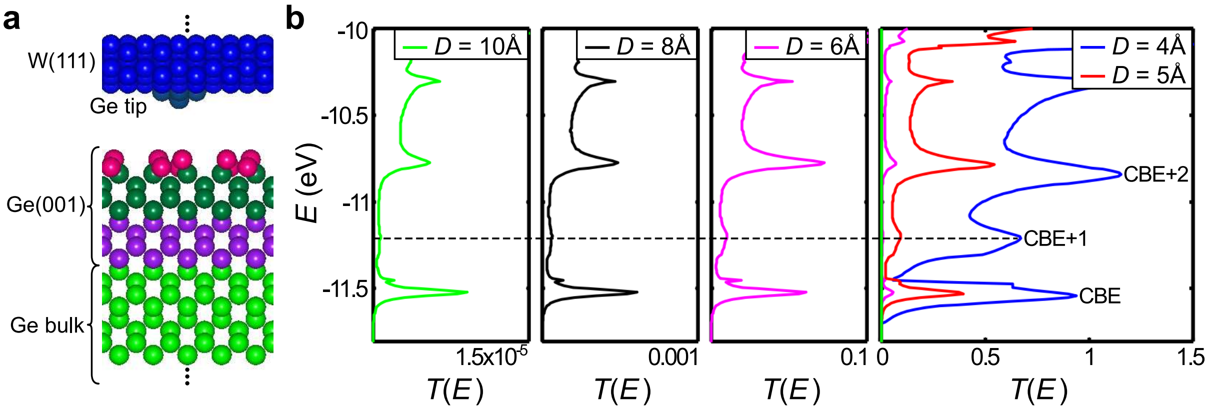

Supplementary Figure 19. **Single probe for different tip-to-surface distances.** (**a**) Scanning tunneling microscopy (STM) configuration setup used for the surface Green-function matching (SGFM) method. (**b**) Transmission function obtained with the simulation setup in **a** for different tip-to-surface distances. A peak corresponding to the CBE+1 becomes clearly visible only at short tip-to-surface distances.

our 3-terminal simulations (**Supplementary Figure 11c**), where the tip-to-surface transmission for different distances present a faster decay for CBE+1 as the tip-sample distance increases.

Supplementary Note 9: k-resolved DOS from Ge(001)-c(4×2): 2-D vs. 1-D character and energy resolved dI/dV maps

In this section we analyze the electronic structure of Ge(001)-c(4×2) away from the high symmetry direction Γ-Y in the Brillouin zone (**Fig. 1a** from the main text). This is relevant to understand both, the level of 1-D character of the electronic propagation along the wires and the Fourier transforms of the experimental dI/dV maps nearby a monatomic step as shown in **Fig. 3** in the main text. The central quantity in our analysis is the $\boldsymbol{k}$-resolved density of states as a function of energy E:

$$\rho(E, k_x, k_y) = \sum_{i=1}^{n} \frac{1}{\pi} \frac{\eta}{(E - \epsilon_n(k_x, k_y))^2 + \eta^2}, \tag{2}$$

where the summation goes over all eigenvalues, k_x and k_y denote wave vector coordinates along the direction perpendicular and parallel to the wires respectively, and η is the broadening parameter (taken in the 0.015-0.02 eV range).

Let us first consider the problem of the energy dependent dI/dV maps as a function of the coordinate perpendicular to the step-egde (y-direction). For simplicity we consider the step as a perfectly reflecting barrier for electrons propagating along the surface, approximate their wave functions by plane waves and use Tersof-Hamman theory. We easily arrive to

$$\frac{dI}{dV}(E, y) = LDOS(E, y) \sim \int dk_y \, |\sin(k_y y)|^2 \, DOS(E, k_y), \tag{3}$$

where

$$DOS(E, k_y) = \int dk_x \, \rho(E, k_x, k_y). \tag{4}$$

Then, for the Fourier transform of the map at a given energy E (omitting an energy dependent constant background) one can get

$$\widetilde{LDOS}(E,q) = \frac{1}{2\pi}\int dy\, LDOS(E,y)\, e^{-iqy} \sim DOS(E,q/2)\,. \qquad (5)$$

In the left panel of **Fig. 3e** in the main text we plot $DOS(E,k_y)$ and compare it with the Fourier transform of the experimental dI/dV maps. In agreement with the experiment, for each energy E we obtain higher intensities for two values of k_y (particularly in the case of the lower CBE surface band). They corresponds to the higher values of $DOS(E,k_y)$, i.e. larger density of states, at the lower (k_y^{max}) and higher (k_y^{min}) energy onsets of the surface bands.

We now proceed to analyze the degree of 1-D character of the band structure of Ge(001)-c(4×2) as a function of the energy. **Supplementary Figure 20** shows this k-resolved DOS evaluated in the first quadrant of the Brillouin zone using 64x64 k-points at selected energies belonging to the energy interval where the empty surface bands reside (between the red dashed lines from **Supplementary Figure 17b**). The surface conduction band edge (CBE) is seen at E ∼0.3 eV (energies given with respect to the valence band edge). In the small energy interval from 0.27 to 0.35 eV, one clearly see that there are regions of the energy isoline where the band gradient in k-space, i.e., the group velocity, has a large component along the k_x direction. Therefore, within this energy interval, electrons can propagate also perpendicularly to the dimer rows, thus, giving rise to a more 2-dimensional character of transport through the surface.

From 0.37 eV until 0.65 eV, on the other hand, the band gradient exhibits small angles with respect to the k_y axis, meaning that the transport is

predominantly along the dimer rows. Such behavior is clearly seen at **Fig. 4f** in the main text, where the incoming states from tip1, evaluated at 0.5 eV and projected into the surface, show a strong localization on a single dimer row.

Starting at CBE+1 (0.67 eV) until 0.75 eV, the transport is again characterized by a 2-dimensional regime. Such behavior may contribute to the observed fast vanishing the interference patterns ($\sim$10 nm) on the step edge experiment for energies $\geq$ 0.7 eV ($\sim$10 nm) as compared to lower energies (**Fig. 3c**, main text). Moreover, it may as well explain the dark feature feature observed in the single-point STS stacking plot (**Fig. 3d**, main text) from 0.7 to 0.8 eV. Finally, our interpretation is in agreement with the simulated eigenchannels on the step-edge setup (**Supplementary Figure 13a**), where right-lead eigenchannels evaluated at 0.29 eV and 0.69 eV exhibit a non-zero probability to propagate through the upper terrace towards the left lead.

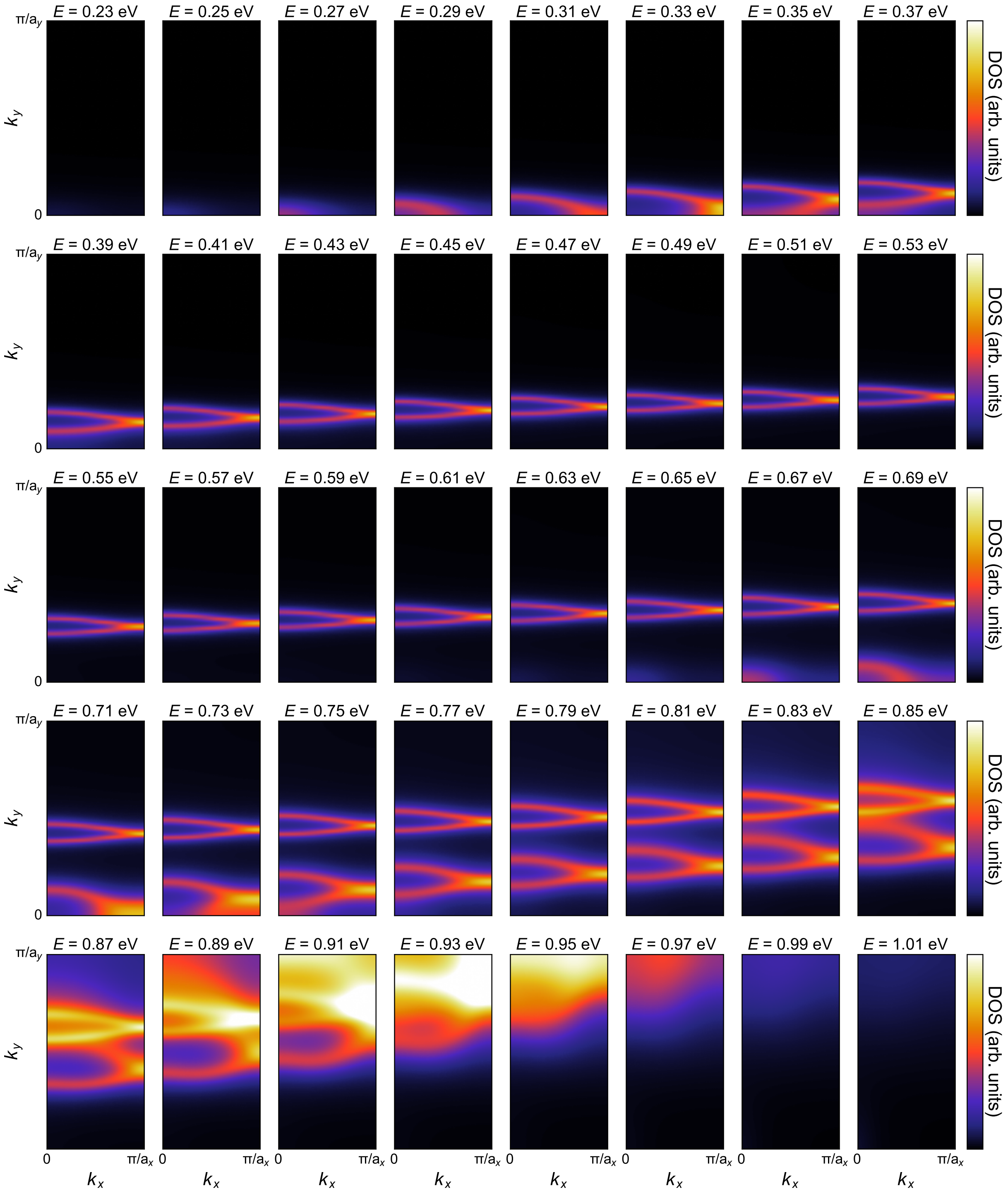

Supplementary Figure 20. **Ge(001)-c(4×2)** k**-resolved density of states.** Density of states of a twelve-layer Ge(001)-c(4×2) slab resolved in the reciprocal space ($\eta = 0.02$ eV) and calculated at selected energies belonging to the empty surface states energy interval.

Supplementary Note 10: Semi-infinite Ge substrate addressed by a single tip

In the transport simulation setups discussed here so far a twelve-layer Ge(001)-c(4×2) slab was used to represent the sample Ge surface. In order to evaluate the effects that a finite slab might introduce in our calculations and to understand how to align the band structures from slab and bulk, we simulate a semi-infinite Ge(001) substrate being approached by a single metallic tip (**Supplementary Figure 21a-b**).

This 2-terminal setup comprises 3934 atoms (31149 orbitals) defined in a supercell of dimensions 32.03×32.03×137.69 Å^3. The same general parameters described in **Sec. 5** were adopt here as well. In this case, however, the Ge electrode is defined by a 8-layer bulk Ge (512 atoms) located 28-layers below the surface (blue box in **Supplementary Figure 21a**). Given the non-periodicity of the the system in the tip-to-bulk direction, more 20-layer Ge were added as buffer (red box in **Supplementary Figure 21a**). The tip-to-surface distance was fixed at D = 4.5 Å and no further geometry optimizations were done, i.e., the Ge surface was fixed by the geometry relaxed without the presence of the tip. This consistent with our previous study, in which we show that relaxations are very small for those large tip-surface separations.

In order to associate the present setup of a full Ge surface with the finite Ge slab calculations, we compared the DOS projected on the surface atoms (blue dashed box in **Supplementary Figure 21a**) with the one obtained from a twelve-layer slab electrode (represented in **Supplementary Figure 10a**), which correspond to the blue and magenta curves in

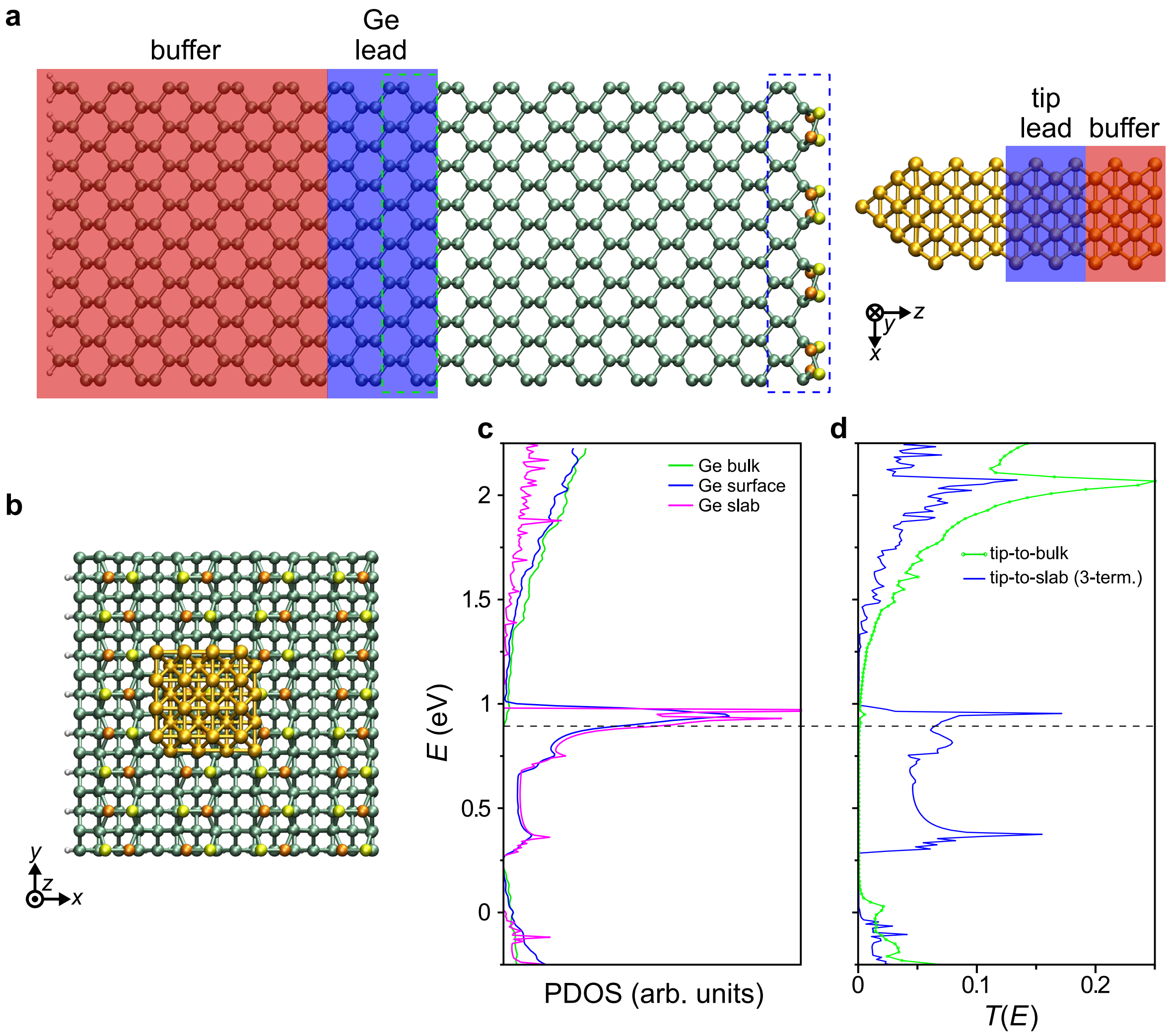

Supplementary Figure 21. **Single metallic tip addressing a full Ge(100)-c(4×2) surface.** (**a**) Side view of the 2-terminal setup comprising a Ge(100)-c(4×2) surface approached by a metallic Au tip. Blue boxes indicate the positions where the infinite electrodes were defined, whereas the red ones highlight the buffer regions included to improve the convergence at the electrode/device interface. Ge buckled dimers are colored in yellow and orange to distinguish them from the other Ge atoms, which are colored in green). (**b**) Top view of the system. (**c**) Calculated DOS projected on bulk Ge (dashed green box in **a**) and surface (dashed blue box in **a**) atoms. Those curves were aligned with respect to the calculated density of states from a twelve-layer Ge slab electrode (right of **Supplementary Figure 10a**) superimposed in magenta. The dashed black line indicates an estimation for the onset of the bulk unoccupied states. (**d**) Transmission function from the Au tip into Ge bulk averaged over the 3×3 transversal k-points (green). The 3-terminal setup discussed in **Sec. 5**, with a single Au tip addressing a Ge slab (**Supplementary Figure 9**, with D = 4.5 Å), was included in the plot (blue) as a reference.

Supplementary Figure 21c, respectively. For consistency, we here also show the results with respect to the pristine Ge slab VBE energy, and shift in energy the surface PDOS calculated for the new system so to match with the slab calculation in the energy range corresponding to the unoccupied surface states (from 0.25 to 1 eV in **Supplementary Figure 21c**). Given the very good agreement between these two curves in the referred energy window, we assume that the surface bands are almost not affected by finite size effects. Therefore, we can apply the same energy shift to the DOS projected on bulk Ge atoms (green dashed box in **Supplementary Figure 21a**), as shown by the green curve in **Supplementary Figure 21c**, which provides an estimation for the onset of the bulk Ge conduction band edge (black dashed line). Accordingly, our analysis indicates that the bulk Ge unoccupied states overlap in energy with CBE+2, with its onset in between the CBE+1 and CBE+2 resonances.

Notice that with this set up, transport calculations only reflect the flow of electrons from the Au tip ontop the bulk states of Ge (i.e., in this case our electrode corresponds to Ge bulk). The calculated transmission function from the Au tip to bulk Ge is presented in **Supplementary Figure 21d**, averaged over the transversal k-points (green). For this elastic transport calculation, no transmission is recorded within the energies corresponding to the bulk Ge gap. Comparing these results with our 3-terminal setup of a Au tip approached to a Ge slab (**Supplementary Figure 9**), we note a very good agreement at higher energies, e.g., the monotonic increment of transmission, and a clear overlap between the onset of the tip-to-bulk transmission with the resonance attributed to CBE+2 in the tip-to-slab transmission.

Supplementary References

[1]Kolmer, M. *et al.* Two-probe STM experiments at the atomic level. *J. of Phys.: Cond. Matt.* **29**, 444004 (2017).

[2]Nakatsuji, K., Takagi, Y., Komori, F., Kusuhara, H. & Ishii, A. Electronic states of the clean Ge(001) surface near Fermi energy. *Phys. Rev. B* **72**, 241308 (2005).

[3]Wojtaszek, M. *et al.* Fermi level pinning at the ge(001) surfacea case for non-standard explanation. *J. Appl. Phys.* **118**, 185703 (2015).

[4]Du, Y. *et al.* Layer-resolved band bending at the $n-$ SrTio$_3$(001)/$p-$ Ge(001) interface. *Phys. Rev. Materials* **2**, 094602 (2018).

[5]Artacho, E., SánchezPortal, D., Ordejón, P., García, A. & Soler, J. M. LinearScaling abinitio Calculations for Large and Complex Systems. *Phys. Stat. Solidi (b)* **215**, 809–817 (1999).

[6]Soler, J. M. *et al.* The SIESTA method for ab initio order-N materials simulation. *J. of Phys.: Cond. Matt.* **14**, 2745 (2002).

[7]Troullier, N. & Martins, J. L. Efficient pseudopotentials for plane-wave calculations. *Phys. Rev. B* **43**, 1993–2006 (1991).

[8]Perdew, J. P. & Zunger, A. Self-interaction correction to density-functional approximations for many-electron systems. *Phys. Rev. B* **23**, 5048–5079 (1981).

[9]Ceperley, D. M. & Alder, B. J. Ground State of the Electron Gas by a Stochastic Method. *Phys. Rev. Lett.* **45**, 566–569 (1980).

[10]Levinshtein, M., Rumyantsev, S. & Shur, M. (eds.) *Handbook Series On Semiconductor Parameters*, vol. 1 (World Scientific, 1996).

[11]Yeyati, A. L., Martín-Rodero, A. & Flores, F. Conductance quantization

and electron resonances in sharp tips and atomic-size contacts. *Phys. Rev. B* **56**, 10369–10372 (1997).

[12]Rangel, T. *et al.* Band structure of gold from many-body perturbation theory. *Phys. Rev. B* **86**, 125125 (2012).

[13]Brandbyge, M., Mozos, J.-L., Ordejón, P., Taylor, J. & Stokbro, K. Density-functional method for nonequilibrium electron transport. *Phys. Rev. B* **65**, 165401 (2002).

[14]Papior, N., Lorente, N., Frederiksen, T., García, A. & Brandbyge, M. Improvements on non-equilibrium and transport Green function techniques: The next-generation TRANSIESTA. *Comp. Phys. Comm.* **212**, 8–24 (2017).

[15]Paulsson, M. & Brandbyge, M. Transmission eigenchannels from nonequilibrium Green's functions. *Phys. Rev. B* **76**, 115117 (2007).

[16]Frederiksen, T., Paulsson, M., Brandbyge, M. & Jauho, A.-P. Inelastic transport theory from first principles: Methodology and application to nanoscale devices. *Phys. Rev. B* **75**, 205413 (2007).

[17]Sagisaka, K. & Fujita, D. Standing waves on Si(100) and Ge(100) surfaces observed by scanning tunneling microscopy. *Phys. Rev. B* **72**, 235327 (2005).

[18]Takagi, Y., Nakatsuji, K., Yoshimoto, Y. & Komori, F. Superstructure manipulation on a clean Ge(001) surface by carrier injection using an STM. *Phys. Rev. B* **75**, 115304 (2007).

[19]Horcas, I. *et al.* WSXM: A software for scanning probe microscopy and a tool for nanotechnology. *Rev. Sci. Instrum.* **78**, 013705 (2007).

[20]Cerdá, J., Hove, M. A. V., Sautet, P. & Salmeron, M. Efficient method for the simulation of STM images. I. Generalized Green-function formalism.

Phys. Rev. B **56**, 15885–15899 (1997).

[21] Kolmer, M. *et al.* Electronic properties of STM-constructed dangling-bond dimer lines on a Ge(001)-(2×1):H surface. *Phys. Rev. B* **86**, 125307 (2012).
